# Selenoprotein W modulates tau homeostasis in an Alzheimer’s disease mouse model

**DOI:** 10.1038/s42003-024-06572-0

**Published:** 2024-07-17

**Authors:** Bingyu Ren, Jiaxin Situ, Xuelian Huang, Qiulong Tan, Shifeng Xiao, Nan Li, Jing Tian, Xiubo Du, Jiazuan Ni, Qiong Liu

**Affiliations:** 1 Department of Anatomy, Neuroscience Laboratory for Cognitive and Developmental Disorders, Medical College of Jinan University, Guangzhou, Guangdong 510630 China; 2 Shenzhen Key Laboratory of Marine Biotechnology and Ecology, College of Life Sciences and Oceanography, Shenzhen University, Shenzhen, Guangdong 518055 China; 3 Shenzhen-Hong Kong Institute of Brain Science-Shenzhen Fundamental Research Institutions Shenzhen, Shenzhen, Guangdong 518055 China

**Keywords:** Alzheimer's disease, Alzheimer's disease, Ubiquitylation

## Abstract

Lower selenium levels are observed in Alzheimer’s disease (AD) brains, while supplementation shows multiple benefits. Selenoprotein W (SELENOW) is sensitive to selenium changes and binds to tau, reducing tau accumulation. However, whether restoration of SELENOW has any protective effect in AD models and its underlying mechanism remain unknown. Here, we confirm the association between SELENOW downregulation and tau pathology, revealing SELENOW’s role in promoting tau degradation through the ubiquitin‒proteasome system. SELENOW competes with Hsp70 to interact with tau, promoting its ubiquitination and inhibiting tau acetylation at K281. SELENOW deficiency leads to synaptic defects, tau dysregulation and impaired long-term potentiation, resulting in memory deficits in mice. Conversely, SELENOW overexpression in the triple transgenic AD mice ameliorates memory impairment and tau-related pathologies, featuring decreased 4-repeat tau isoform, phosphorylation at Ser396 and Ser404, neurofibrillary tangles and neuroinflammation. Thus, SELENOW contributes to the regulation of tau homeostasis and synaptic maintenance, implicating its potential role in AD.

## Abstract (web summary)

A study shows that SELENOW binds tau and promotes its degradation via the ubiquitin‒proteasome system. SELENOW deficiency causes synaptic defects and memory deficits in mice, while overexpression in AD models has alleviating effects, highlighting its potential role in AD.

## Introduction

Tauopathies are a series of neurodegenerative diseases that share the same hallmark of accumulating abnormal microtubule-associated protein tau (MAPT). Alzheimer’s disease (AD), the most common dementia in the aging population, is a complex and heterogeneous neurodegenerative disorder characterized by tauopathy and amyloidogenesis. These misfolded and accumulated proteins, such as tau tangles and amyloid-β (Aβ) deposits, result in synaptic dysfunction and neuronal loss in brain regions, eventually lead to progressive cognitive decline, memory loss, and impaired executive function. Increasing evidence indicates that misfolded protein clearance and protein balance restoration may contribute to the alleviation of AD pathologies^1^. As an essential trace element, selenium is particularly well maintained in the brain^2^. In AD brains, decreased selenium levels have been reported^3^ in both soluble and insoluble fractions, as well as associations with the ApoE *ε*4 allele^4^. Treatments with selenium, whether in organic or inorganic form, improve memory decline and alleviate tau-related pathological symptoms in AD models^5–9^. Selenoprotein W (SELENOW) is a small protein with a highly conserved thioredoxin (TXN)-like motif (CXXU) containing a selenocysteine residue^10^. As one of the selenium-sensitive selenoproteins, SELENOW has been found to be enriched in both mouse and human brain^11,12^ and particularly widespread in neurons and dendritic processes^13^. Under selenium deficiency conditions, the level of SELENOW is the last to decrease among all selenoproteins in the brain, suggesting its important role in brain protection^14^. In our previous study, we discovered that SELENOW can bind to tau via disulfide linkage in vitro and attenuate tau accumulation in HEK293 cells^15^. Taken together, these studies support the hypothesis that SELENOW may be a target for selenium treatment in AD by relieving tau-related pathology. At present, whether restoration of SELENOW’s level has any relieving effect on AD and the mechanism of how it is involved in tau regulation remain unknown. The ubiquitin‒proteasome system (UPS) and autophagy have been considered the two major degradation pathways for tau clearance^16^. Previous studies reported that SELENOW could control the degradation of epidermal growth factor receptors via ubiquitination in epithelial cell lines^17^, and knockout of SELENOW resulted lower levels of Yap1 ubiquitination in mouse colon^18^. Correspondingly, in this study we found that by competing with Hsp70, SELENOW promoted tau ubiquitination levels and inhibited acetylation at K281. SELENOW knockout induced tau dysregulation, as well as synaptic and memory deficits in the mouse brain. In turn, SELENOW overexpression mitigated memory impairment and tau-related pathologies in the triple transgenic (3×Tg) AD mouse model, indicating a potential protective effect of SELENOW.

## Results

### SELENOW levels were inversely correlated with tau

**Fig. 1:**
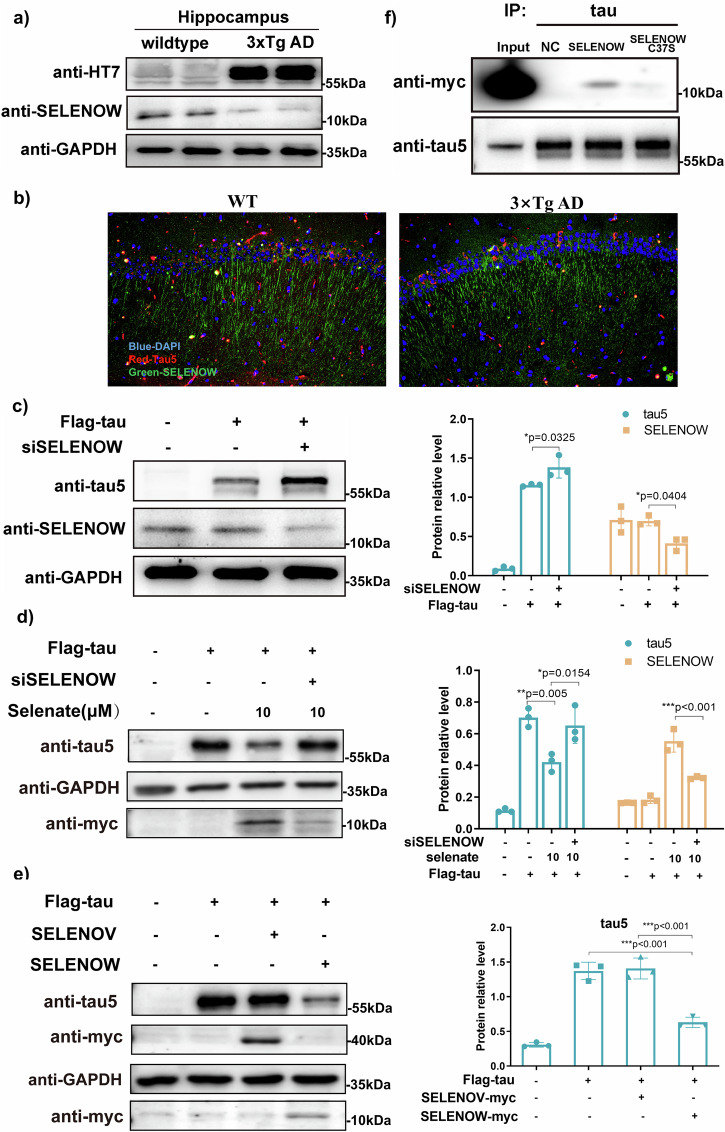
SELENOW levels were negatively correlated with tau expression levels. **a** Representative western blots of the 8-month-old WT and 3×Tg AD mouse hippocampus, showing the typical overexpression of tau protein and downregulation of SELENOW in AD. **b** Representative immunostaining of SELENOW (green), tau5 (red) and cell nuclei (blue) in the hippocampal CA1 areas of 8-month-old WT and 3×Tg AD mice. Scale bar = 200 μm. **c** HEK293TAU cells were transiently transfected with scramble or SELENOW siRNA. Representative immunoblot showing that the knockdown of SELENOW by siRNA upregulated the expression of tau. **d** HEK293TAU cells were transiently transfected with scramble or SELENOW siRNA and then treated with selenate for 24 h. Representative western blot showed that selenium supplementation by selenate induced the upregulation of SELENOW and reduced the expression of tau. Knockdown of SELENOW blocked selenate-induced tau downregulation. **e** HEK293TAU cells were transiently transfected with SELENOV or SELENOW. Representative western blot showed that the overexpression of SELENOW reduced the expression of tau while overexpression of SELENOV had no effect on tau expression. **f** HEK293TAU cells were transiently transfected with SELENOW or its C37S mutant, the same amounts of tau were immunoprecipitated, and the samples were subjected to western blot analysis. Representative results showed that SELENOW immunoprecipitated with tau, while C37 mutated to S in SELENOW diminished their interaction. Data are presented as the mean ± SD. *N* = 3 in each group, *p < 0.05, **p < 0.01 and ***p < 0.001 by one-way ANOVA.

According to Alzdata’s expression profiling in Supplementary Fig. 1a, SELENOW was significantly downregulated in the hippocampus and temporal cortex of AD patients^19^. In our former study, we discovered that as SELENOW increased, tau proteins decreased^15^, indicating a negative relationship between tau pathology and SELENOW. To further address their relationship, levels of tau and SELENOW were measured in AD cell and mouse models. Immunoblots showed reduced SELENOW in the hippocampi of 8-month-old 3×Tg AD mice compared with wild-type (WT) mice (Fig. 1a). Mouse brain immunostaining indicated that SELENOW was enriched in the axonal processes of hippocampal CA1 neurons in WT mice (Fig. 1b left panel). Similar to the blot results, obvious loss of SELENOW in AD cases was detected. In tau stably expressed HEK293 cells (HEK293TAU), downregulation of SELENOW by siRNA resulted in a further increase in tau (Fig. 1c), while upregulation of SELENOW by selenate treatment resulted in a reduction in tau (Fig. 1d and Supplementary Fig. 1b). Conversely, tau increased when the upregulation of SELENOW by selenate was neutralized by siRNA. Furthermore, we used another cytoplasmic selenoprotein, SELENOV, which also possesses a TXN-like motif, to investigate whether it exhibits similar regulatory effects on tau as SELENOW. The results in Fig. 1e demonstrated that the overexpression of SELENOV had no effect on the levels of tau protein, suggesting that the regulatory effect on tau was not universal among all members of selenoproteins. We previously identified a disulfide linkage between cysteine-37 (C37) of SELENOW and cysteine-322 (C322) of tau in vitro, which might be the key point for the reduction of tau protein induced by SELENOW^15^. The co-IP results in Fig. 1f further confirmed the binding between SELENOW and tau. Substitution of SELENOW C37 with serine (C37S) mainly abolished its binding ability to tau, indicating that SELENOW C37 was crucial for mediating their interaction.

### SELENOW downregulated tau protein levels by promoting its degradation

**Fig. 2:**
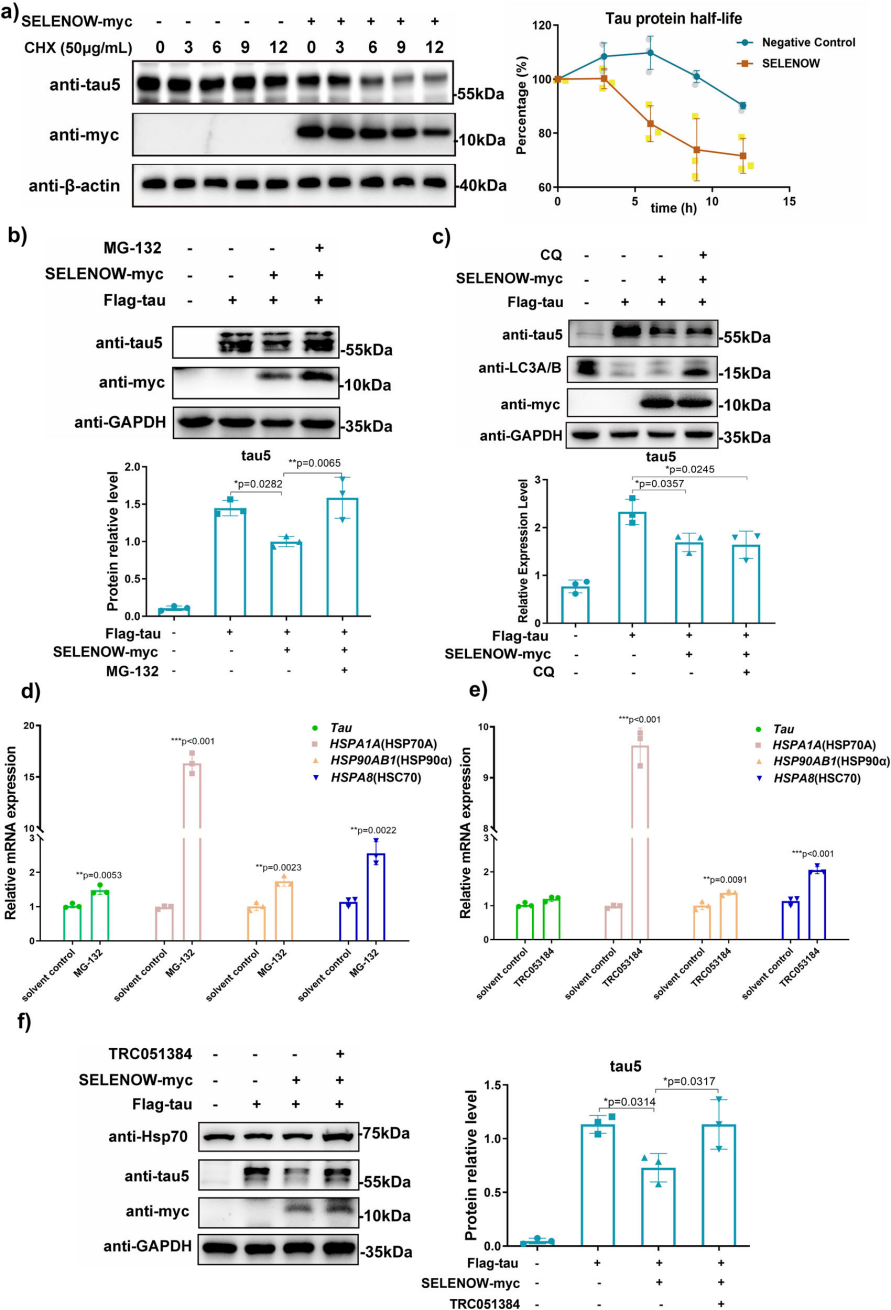
SELENOW induced tau protein clearance through the ubiquitin‒proteasome system. **a** HEK293TAU cells were transiently transfected with empty plasmids or SELENOW and then treated with the protein synthesis inhibitor CHX. The samples were collected at several time intervals and subjected to western blot analysis. Protein levels showed accelerated half-life of tau protein by SELENOW overexpression. Protein levels of β-actin were used as a control for normalization. **b** HEK293TAU cells were transiently transfected with empty plasmids or SELENOW and then treated with the 20 S proteasome inhibitor MG-132 for 6 h. Representative western blot showing that MG-132 blocked SELENOW-induced tau clearance. **c** HEK293TAU cells were transiently transfected with empty plasmids or SELENOW and then treated with the autophagy inhibitor CQ for 6 h. Representative immunoblot showing that CQ had no effect on SELENOW-induced tau clearance. The transcription levels of MAPT and heat shock proteins were quantified by Q-PCR in HEK293TAU cells treated with (**d**) MG-132 and (**e**) the heat shock protein inducer TRC051384. ACTB was used as an endogenous reference gene. **f** HEK293TAU cells were treated with TRC051384, and the samples were collected for western blotting. Representative immunoblots showed that TRC051384 reversed the effect of SELENOW on tau protein degradation. Data are presented as the mean ± SD. *N* = 3 in each group, *p < 0.05, **p < 0.01 and ***p < 0.001 by *t* tests between two groups, and by one-way ANOVA for groups that had more than two.

With a negative correlation between SELENOW and tau levels established, the effect of SELENOW on tau homeostasis, especially tau degradation, was further explored. First, the protein half-life of tau was analyzed by the cycloheximide (CHX) chase assay. CHX is an inhibitor of new protein synthesis, as it may prevent translational elongation. Comparison of protein stability (or degradation rate) in eukaryotic cells has been achieved by chasing the protein level changes at specific time points following the addition of 50 μg/mL CHX^20^. Compared with the negative control, acceleration of tau degradation was observed in the presence of SELENOW (Fig. 2a), indicating that the interaction of SELENOW and tau seemed to promote the cellular clearance of tau. There are two main pathways for pathological tau degradation: UPS and autophagy. Next, we used chemical inhibitors for these two pathways separately. Application of the 20 S proteasome inhibitor MG-132 successfully blocked SELENOW-mediated tau clearance (Fig. 2b), while application of the autophagy inhibitor chloroquine (CQ) had no significant effect (Fig. 2c). Thus, overexpression of SELENOW may accelerate tau degradation via the ubiquitin‒proteasome pathway instead of autophagy. The molecular chaperone heat shock proteins have been proven to be functional in the fate determination of tau, which is to be retained or to be degraded^21^. We found that MG-132 treatment induced the upregulation of *MAPT* mRNA, as well as several heat shock proteins (Fig. 2d). Then, the chemical heat shock protein inducer TRC053184 was applied, which increased the mRNA levels of heat shock proteins without any significant impact on tau mRNA (Fig. 2e). Similar to the result of MG-132, treatment with TRC053184 also antagonized the effect of SELENOW overexpression on tau levels (Fig. 2f).

### SELENOW interacted with tau in competition with Hsp70, promoted the ubiquitination of tau, and affected its acetylation at the K281

**Fig. 3:**
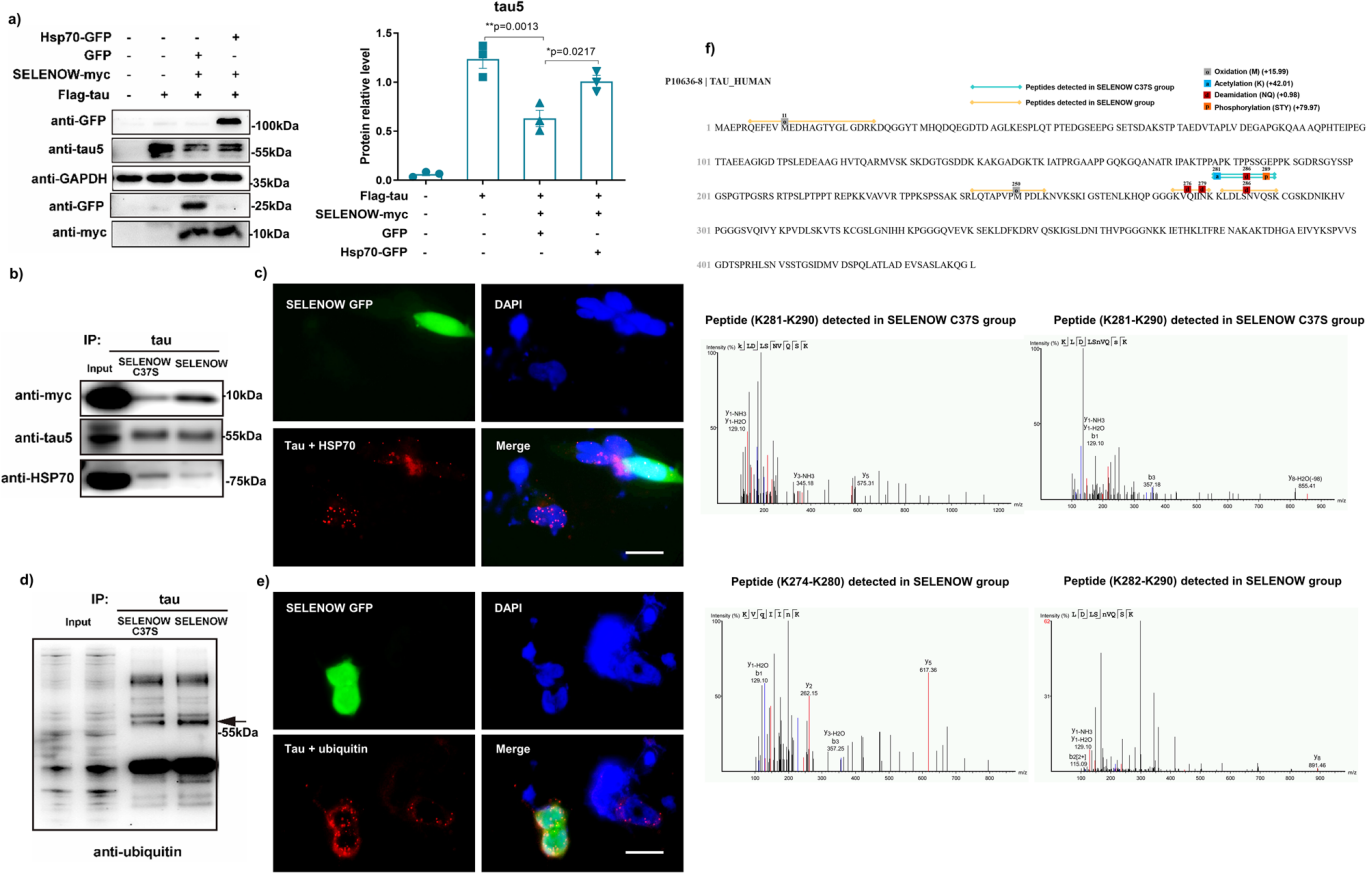
SELENOW competed with Hsp70 for tau binding, inhibited tau acetylation and promoted tau polyubiquitination. **a** HEK293TAU cells were transiently transfected with GFP or Hsp70-GFP, and the samples were collected for western blotting. Representative immunoblots showed that HSP70 overexpression partially antagonized the effect of SELENOW on tau protein degradation. HEK293TAU cells were transiently transfected with SELENOW or its C37S mutant, and the same amounts of tau were immunoprecipitated. Representative Western blots of immunoprecipitated samples showing (**b**) Hsp70 was released from tau while SELENOW binding increased, and (**c**) the SELENOW overexpression group exhibited increased ubiquitination of tau compared to its C37S mutant. The arrow indicates the predicted band size of tau. HEK293TAU cells were transiently transfected with SELENOW GFP for 48 h, and then PLA fluorescence detection was performed. Representative images showing that (**d**) SELENOW GFP potently decreased the Hsp70-tau complex and (**e**) promoted tau ubiquitination compared with adjacent nontransfected cells. Scale bar = 20 μm. **f** LC/MS-MS detection of soluble tau proteins immunoprecipitated from HEK293TAU cells that overexpressed SELENOW or the C37S mutant. Upper panel: Schematic diagram of tau sequence and modifications detected specifically in the SELENOW C37S or SELENOW group. Lower panel: peptide fragments from K274 to K290 detected by mass spectrometric analysis in SELENOW C37S group and SELENOW group. Data are presented as the mean ± SD. *N* = 3 in each group, *p < 0.05, **p < 0.01 and ***p < 0.001 by one-way ANOVA.

During the chaperone-mediated tau degradation process, the importance of Hsp70 was highlighted by numerous previously reported works^22–24^. Our Q-PCR results suggested that its mRNA increased predominantly in both MG-132 and TRC053184 treatments. Therefore, we further assessed the effect of Hsp70 upregulation on SELENOW-mediated tau degradation. As expected, overexpressing Hsp70-GFP attenuated SELENOW-mediated tau degradation compared to the GFP negative control (Fig. 3a). During protein quality control, the Hsp70 family and their cochaperones form a complex with tau to regulate its stability^1^. Two Hsp70 binding motifs identified by NMR experiments within the microtubule binding region of tau, the V275 to N279 and V306 to Y310 segments, are close to the residue that mediates SELENOW and tau interactions. We speculated that SELENOW may compete with Hsp70 for binding sites on tau. Indeed, our results showed decreased binding of Hsp70 to tau in cells overexpressing SELENOW (Fig. 3b). Proximity ligation assay images confirmed fewer Hsp70-tau complexes in SELENOW-GFP cells compared to adjacent non-transfected cells (Fig. 3c).

In addition to our experimental findings, we employed the ComplexContact^25^ to forecast the potential contact residues within the SELENOW-tau and Hsp70-tau complexes (Supplementary Fig. 2). The projected tau segment for SELENOW binding spanned from S305 to C322, while that for Hsp70 binding spanned from S305 to Y310. The prediction for Hsp70 was in accordance with the aforementioned known motif, and the C322 site identified through our experimentation was also encompassed in the SELENOW prediction results, indicating that the predictions made by ComplexContact exhibit a high degree of reliability. A previous study reported that interference with the Hsp70-tau complex might promote the polyubiquitination of tau which leads to tau clearance^26^. Correspondingly, we observed increased levels of tau ubiquitination in cells overexpressing SELENOW compared to SELENOW C37S (Fig. 3d). Besides, we also visualized more tau ubiquitination signal in situ in SELENOW-GFP cells than in nearby cells (Fig. 3e).

Both ubiquitination and acetylation of tau mainly occur on the lysine residues in the MTBR. The C322 site is important for the interaction between SELENOW and tau, as well as tau autoacetylation^27^. Additionally, lysine residues that can be ubiquitinated are also potential sites for acetylation, suggesting a possible competition between these two post-translational modifications (PTMs)^28^. Thus, ICP‒MS/MS were applied to detect changes in tau PTMs induced by SELENOW overexpression. The peptides in the SELENOW C37S and SELENOW groups shared the same PTMs in most cases, except for peptides from K274 to K290 and peptides that contained M11 and M250 residues (Fig. 3f). Acetylation at K281, deamidation at N286 and phosphorylation at S289 in tau were found in the SELENOW C37S group, while only deamidation at Q276, N279 and N286 was detected in the SELENOW group. This result indicated that binding of SELENOW at C322 may inhibit tau acetylation at K281. In addition, oxidations at M11 and M250 were identified in the SELENOW group (Supplementary Fig. 3).

### SELENOW deficiency induced synaptic defects, tau dysregulation and memory deficits in mice

**Fig. 4:**
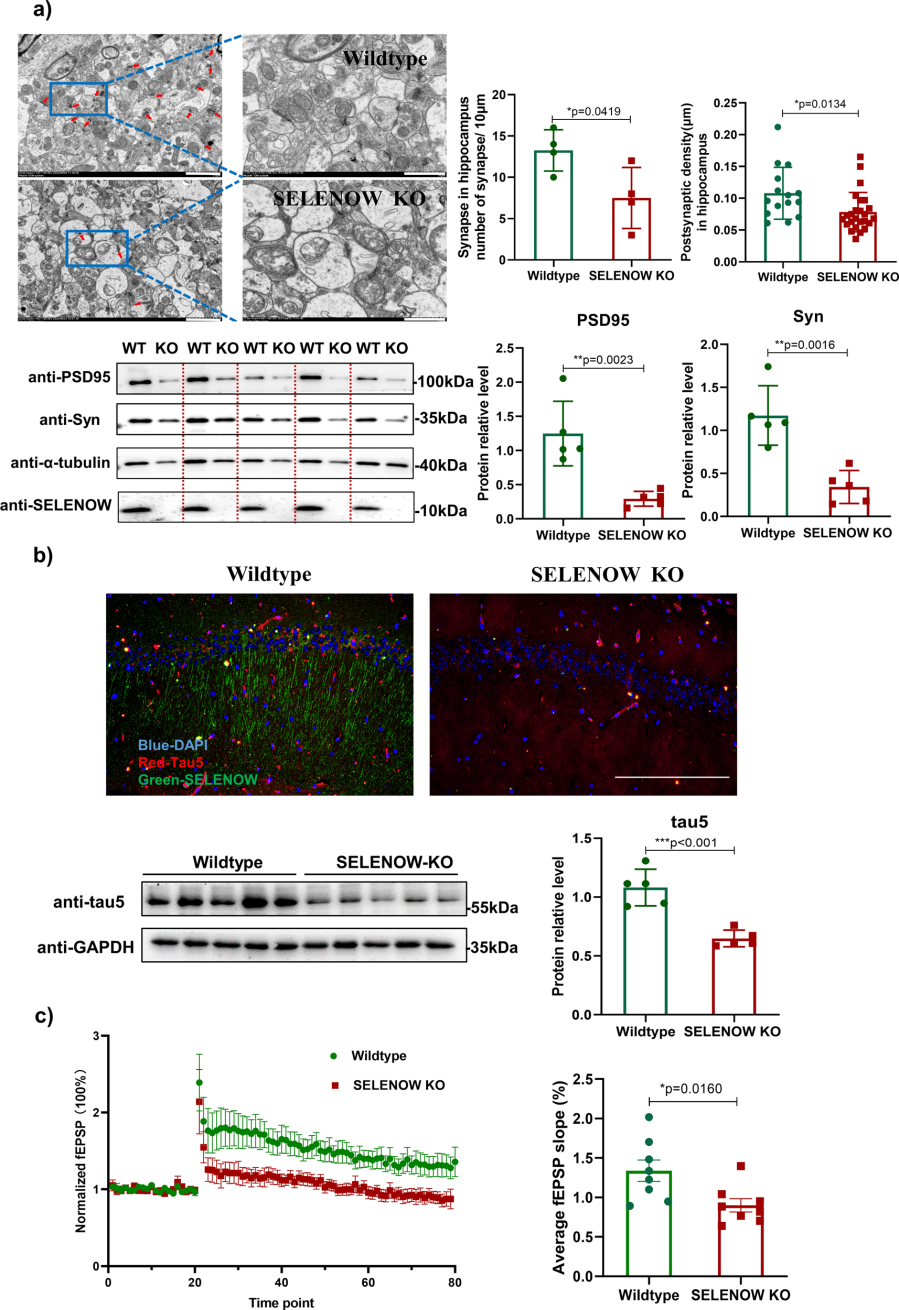
SELENOW deficiency induced synaptic deficits and tau downregulation in the mouse hippocampus. **a** Reduced number of synapses and postsynaptic density in the hippocampus of 6-month-old SELENOW KO mice. Representative and quantification of transmission electron microscope (TEM) images showing the synapse distribution (left panels, as indicated by the red arrows) and ultrastructure (right panels, magnified images) in the cornu ammonis 1 (CA1) region of WT and SELENOW KO mice. Scale bar = 2.0 μm. Representative immunoblots showed a significant decrease in PSD95 and the synaptic vesicle marker Syn in the hippocampus of SELENOW KO mice. **b** Downregulation of tau protein levels in the hippocampus of 6-month-old SELENOW KO mice. Representative immunostaining images of SELENOW (green), tau5 (red) and cell nuclei (blue) in the hippocampal CA1 areas of 6-month-old WT and SELENOW KO mice. Scale bar = 200 μm. Representative immunoblots showed significant downregulation of tau in the hippocampus of SELENOW KO mice. **c** Impaired LTP recordings in the hippocampus of 6-month-old SELENOW KO mice. Slope of fEPSPs in response to 100-Hz stimulation in the Schaffer collateral CA1 region of WT and SELENOW KO mice (left panel). Quantification of the averaged fEPSP slope (right panel). Representative traces 20 min before and 60 min after θ-burst stimulation are shown. Data are presented as the mean ± SD. *N* = 4 slices in each group for synapse number analysis, *N* = 15 synapse from 3 mice in each group for postsynaptic density analysis, *N* = 5 from 5 mice in each group for immunoblots, and *N* = 8 slices from 6 mice for LTP recording, *p < 0.05, **p < 0.01 and ***p < 0.001 by t test.

To determine whether the existence of SELENOW was important for neuronal function and tau regulation, conventional SELENOW knockout (KO) mouse was used. Features of synapse ultrastructure and markers of synaptic proteins were evaluated at the age of 6 months. The SELENOW KO mice exhibited fewer synapses and decreased postsynaptic density in the hippocampal CA1 region (Fig. 4a). Additionally, immunoblotting detected a reduction in postsynaptic density protein 95  (PSD95) and synaptic vesicle marker synaptophysin (Syn) proteins in the hippocampus of SELENOW KO mice. Immunostaining and immunoblotting detected decreased tau protein in the hippocampus of SELENOW KO mice (Fig. 4b). It is known that changes in synaptic density are typically associated with altered synaptic function. Also, tau is essential for long-term potentiation and depression in the hippocampus^29,30^. Thus, we investigated the effect of SELENOW deficiency on synaptic transmission and plasticity in hippocampal slices. SELENOW KO mice exhibited a downward shift in the field excitatory postsynaptic potential (fEPSP) slope compared to WT mice, and LTP induction was significantly reduced (Fig. 4c).

**Fig. 5:**
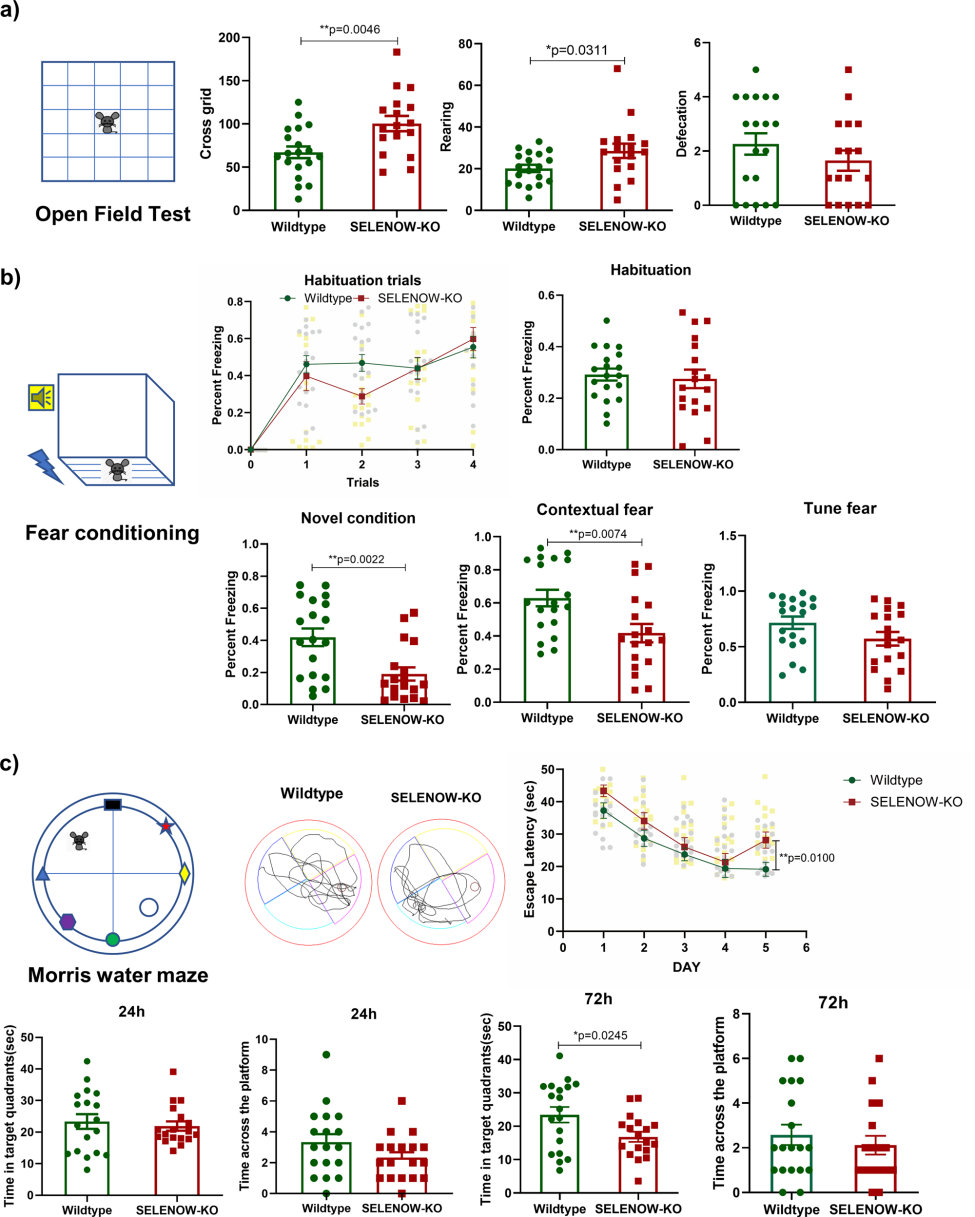
SELENOW KO mice exhibited less anxiety-like behavior and impaired memory. **a** Locomotion ability and anxiety of the 12-month-old WT and SELENOW KO mice evaluated by the crossing grid numbers, rearing and defecation times in the open field test. **b** Fear memory of the 12-month-old WT and SELENOW KO mice evaluated by the percent freezing in habituation, contextual fear, novel condition, and tune fear stages in the contextual fear memory task. Representative track trails of WT and KO mice in the contextual fear stage are also provided. **c** Spatial and working memory of the 12-month-old WT and SELENOW KO mice evaluated by the escape latency, the time the mice spent in the target in 24 h and 72 h trials, and the number of times the mice crossed the platform quadrant in 24 h and 72 h trials of the Morris water maze. The representative track trails of WT and KO mice in the 72 h trials are also provided. Data are presented as the mean ± SD. *N* = 18 in each group, outliers were detected and excluded using the Grubbs’ test, *p < 0.05, **p < 0.01 and ***p < 0.001 by t test.

Changes at the synaptic level are likely to underlie subtle memory impairments with AD-like phenotypes. Indeed, we previously reported less anxiety-like behavior and impaired contextual fear memory in 6-month-old female SELENOW KO mice, whereas their spatial working and learning memories seemed to be reserved at this age^31^. Given that evident memory deficits in AD models usually occur at an older age, we also investigated the behavioral changes in SELENOW KO mice at 12 months of age. Similar to our former results from the 6-month-old mice, our 12-month-old SELENOW KO mice demonstrated a significant increase in the number of grid crossings and rearing times in the OFT compared with their age-matched WT (Fig. 5a). They also showed an obvious decrease in the freezing percentage in the contextual fear memory tests, with the addition of freezing percentage reduction during novel condition fear memory tests (Fig. 5b). Moreover, the 12-month-old SELENOW KO mice exhibited a significantly longer latency on day 5 to reach the hidden platform than WT mice during navigation testing in the Morris water maze. In the probe trial test, 12-month-old SELENOW KO mice spent less swimming time in the target quadrant 72 h after the last training on the 5th day (Fig. 5c), suggesting that learning and memory deficits emerged in SELENOW KO mice at 12 months of age.

### SELENOW overexpression ameliorated memory deficits in 3×Tg AD mice

**Fig. 6:**
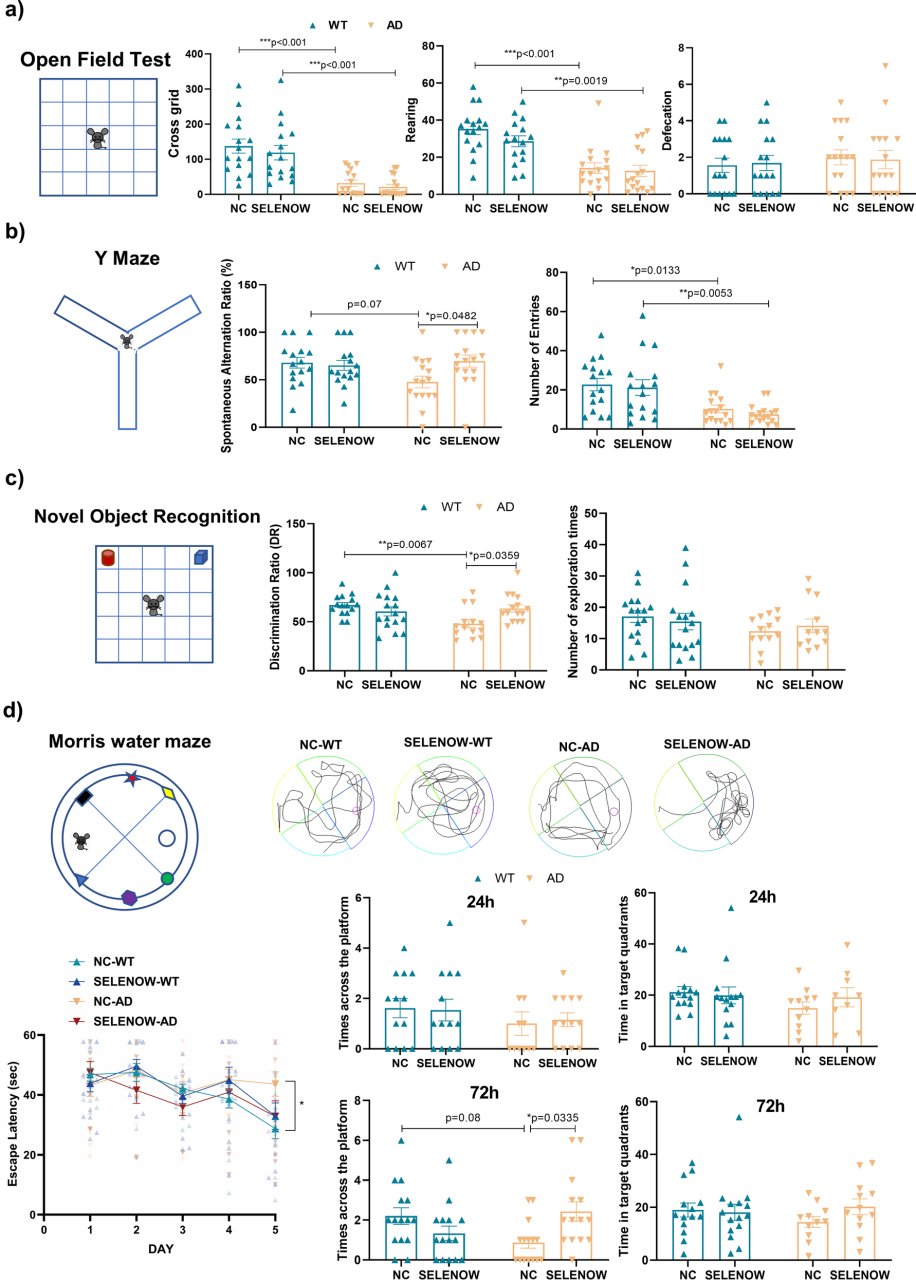
Brain SELENOW overexpression rescued behavioral deficits in 3×Tg AD mice. **a** Locomotion ability and anxiety of 12-month-old WT and 3×Tg AD mice receiving empty vector (NC-WT and NC-AD) or AAV-SELENOW (SELENOW-WT and SELENOW-AD) in their hippocampal CA3 area, evaluated by the number of grid crossings and rearing and defecation times in the open field test. **b** Spatial memory of the 12-month-old NC-WT, SELENOW-WT, NC-AD and SELENOW-AD mice evaluated by the spontaneous alternation ratio and number of total arm entries in the Y-maze task. **c** Declarative memory of the 12-month-old NC-WT, SELENOW-WT, NC-AD and SELENOW-AD mice evaluated by the discrimination ratio and total times in the novel object recognition task. **d** Spatial and working memory of the 12-month-old NC-WT, SELENOW-WT, NC-AD and SELENOW-AD mice evaluated by the escape latency, the time the mice spent in the target in 24 h and 72 h trials, and the number of times the mice crossed the platform quadrant in 24 h and 72 h trials of the Morris water maze. Representative track trials in the 72 h trials are also provided. Data are presented as the mean ± SD. *N* = 18 in each group, outliers were detected and excluded using the Grubbs’ test, *p < 0.05, **p < 0.01 and ***p < 0.001 by two-way ANOVA.

To further address the possible protective role of SELENOW in the AD brain, the effects of SELENOW overexpression were assessed in WT and 3×Tg AD mouse models. Six-month-old WT and 3×Tg AD mice were randomly injected with AAV packed empty vector as a negative control (NC-AD) or SELENOW(U13C) overexpression vector (SELENOW-AD) in their hippocampal CA3 region. The overall expression of GFP in the hippocampus was successfully detected two months after the injection (Supplementary Fig. 4). Six months after the injection, the mice were initially assessed in the behavioral tests. Generally, AD mice showed decreased motor activities compared with WT mice, regardless of whether receiving empty vector or treated with AAV-SELENOW, as evidenced by significant reduction of the number of grid crossings and rearing times in OFT, and total number of arm entries in Y maze (Fig. 6a, b). Furthermore, the NC-AD mice also exhibited memory deficits compared to NC-WT mice, with a significant decrease of discrimination ratio in novel object recognition tests (Fig. 6c), and a longer latency on day 5 to reach the hidden platform in Morris Water Maze (Fig. 6d).

The SELENOW-AD mice demonstrated no obvious change in the number of grid crossings, rearing and defecation times in the OFT and no obvious change in the tendency to explore the open arms in the elevated plus maze compared with NC-AD mice (Fig. 6a and Supplementary Fig.5a). In Y maze and novel object recognition tests, the SELENOW-AD mice exhibited an increased spontaneous alternation ratio and discrimination ratio compared with NC-AD, with no difference in the total number of arm entries and exploration times (Fig. 6b, c). In Morris water maze, the SELENOW-AD mice spent more swimming time in the target quadrant in the 72 h probe trial test than the NC-AD (Fig. 6d). Together, these results suggested that the overexpression of SELENOW ameliorated the learning and memory deficits in 3×Tg AD models without altering their locomotion ability and anxiety level.

### SELENOW overexpression modulated tau-related pathology and neuroinflammation in 3×Tg AD mice

**Fig. 7:**
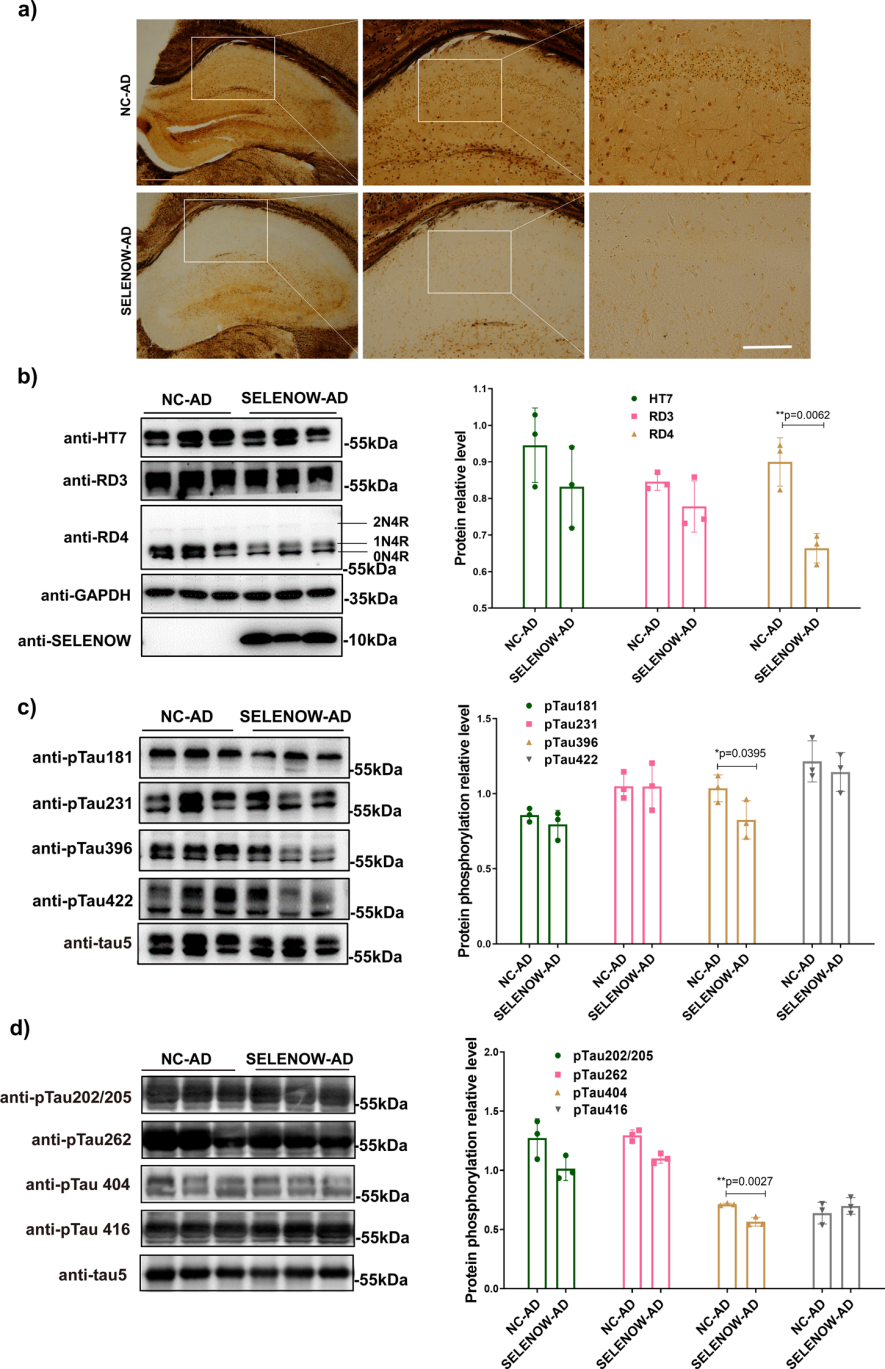
Brain SELENOW overexpression reduced tau pathologies in 3×Tg AD mice. **a** Bielschowsky silver staining of neurofibrillary tangles in the hippocampus of 12-month-old NC-AD and SELENOW-AD mice. Scale bar = 500 μm. **b** Representative western blot results and analysis of soluble total tau (HT7), 3-repeat isoform tau (RD3), and 4-repeat isoform tau (RD4) in brain hippocampus homogenates from 12-month-old NC-AD and SELENOW-AD mice. **c** Representative western blot results and analysis of tau phosphorylation at different sites, including Thr181, Thr231, Ser396 and Ser422 in brain hippocampus homogenates from 12-month-old NC-AD and SELENOW-AD mice. **d** Representative western blot results and analysis of tau phosphorylation at different sites, including Ser202/Thr205, Ser262, Ser404 and Ser416 in brain hippocampus homogenates from 12-month-old NC-AD and SELENOW-AD mice. The relative protein phosphorylation levels were defined as phosphorylated tau normalized against total tau (tau5). Data are presented as the mean ± SD. *N* = 3 in each group, *p < 0.05, **p < 0.01 and ***p < 0.001 by *t* tests.

Two weeks after the behavioral tests, the mice were euthanized for assays of pathological changes. Since the above results indicated that SELENOW may be involved in maintaining tau homeostasis, we first focused on changes in tau-related pathological indicators. The effect of SELENOW overexpression on the formation of neurofibrillary tangles was detected by silver staining (Fig. 7a). Remarkably, the density of the Alzheimer-like neurofibrillary tangles was reduced in the hippocampus of SELENOW-AD mice. The immunoblots showed that compared with NC-AD, SELENOW-AD successfully expressed the SELENOW protein at a high and steady level, and had a significant reduction in the levels of the 4-repeat tau isoform, while we did not observe any significant change in the levels of total tau and the 3-repeat tau isoform (Fig. 7b). As excessive tau phosphorylation disrupts its function and structure, promoting its aggregation into tangles, we further assessed changes of tau phosphorylation at different epitopes. According to the results in Fig. 7c, d, SELENOW-AD mice had a significant decrease in tau phosphorylated at epitope Ser396 and Ser404, but no changes were observed for tau phosphorylated at epitopes Thr181, Ser202/Thr205, Thr231, Ser262, Ser416 and Ser422.

**Fig. 8:**
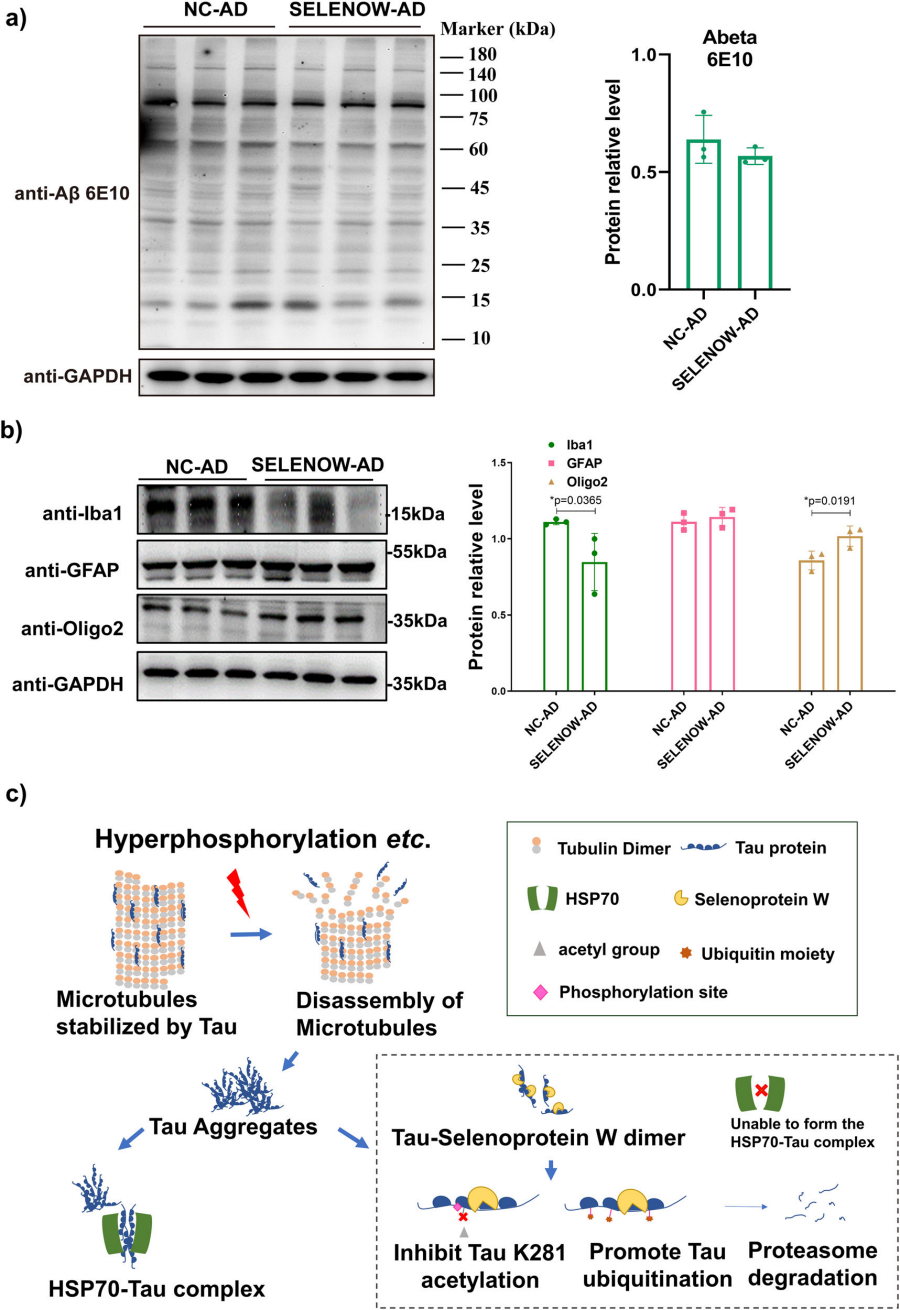
Brain SELENOW overexpression alleviate neuroinflammation in 3×Tg AD mice. **a** Representative western blot results and analysis of Aβ oligomers detected with 6E10 antibody in brain hippocampus homogenates from 12-month-old NC-AD and SELENOW-AD mice. **b** Representative western blot results and analysis of neuroglial cell markers, including microglia (Iba-1), astrocyte (GFAP) and oligodendrocyte (Oligo2), in brain hippocampus homogenates from 12-month-old NC-AD and SELENOW-AD mice. **c** A model for SELENOW overexpression in AD promoted the degradation of abnormal tau. SELENOW formed heterodimers with tau via C322, which also mediated tau’s interaction with HSP70 and its auto-acetylation. The interaction of SELENOW and tau affected the formation of the HSP70-tau complex, inhibited tau acetylation at K281, and influences phosphorylation at adjacent sites of Ser396 and Ser404. Non-acetylated lysine sites of tau were more likely to undergo polyubiquitination, leading to proteasomal degradation of abnormal tau. Data are presented as the mean ± SD. *N* = 3 in each group, *p < 0.05, **p < 0.01 and ***p < 0.001 by *t* tests.

Aβ and tau pathologies are closely intertwined in the progression of AD. Immunoblot of Aβ using 6E10 antibody was conducted. As demonstrated in Fig. 8a, no significant change had been detected between NC-AD and SELENOW-AD groups. Besides, it is known that memory impairments in AD are typically associated with alterations in oxidative stress and neuroglia. Thus, MDA and markers for microglia (Iba-1), astrocytes (GFAP) and oligodendrocytes (Oligo2) were assessed. As shown in Supplementary Fig. 5b, no significant change of MDA levels had been detected, while in Fig. 8b, SELENOW overexpression significantly reduced the levels of Iba-1 and upregulated the levels of Oligo2 in 3×Tg AD mice. Given the above, these results revealed that the overexpression of SELENOW in the brain alleviated tau-related pathologies in 3×Tg AD models, with a reduction in neuroinflammation levels.

## Discussion

Numerous reports have proven that restoration of selenium levels in the AD brain can alleviate AD pathology^6,32,33^. The biological functions of selenium are mostly exerted through selenoproteins, some of which are strongly associated with maintaining optimal brain function^34^. The levels of SELENOW ranked the highest among all selenoproteins in the brain^35^, and were broadly expressed across multiple neuronal and non-neuronal cell types^12^. Functional studies from the genetic knockout SELENOW mice primarily focused on its function in bone and liver^36–38^. However, unlike the high-profile brain function-associated selenoproteins, such as SELENOP, GPX1, and GPX4, the brain function of SELENOW and its possible involvement in AD were greatly neglected. In this study, we demonstrated that SELENOW, the smallest selenoprotein known for its antioxidant capacity^2^, played a protective role in the brain.

In cell models, SELENOW upregulation induced a significant reduction in tau via UPS. The mice lacking of SELENOW protein may cause synaptic defects, tau dysregulation and memory impairment. In turn, the AAV mediated gene transfer approach was applied to overexpressing SELENOW in the hippocampus of the 3xTg mice exhibiting Aβ-tau neuropathology and behavioral deficits. SELENOW overexpression rescued behavioral deficits, with amelioration of tau-related pathology. Nevertheless, we did not detect obvious impact of SELENOW overexpression on Aβ pathology in the hippocampus. The observation of Aβ aligns with the widely accepted AD hypothesis, wherein extracellular aggregation and deposition of Aβ seems to serve as the initial pathogenic event, subsequently inducing tau hyperphosphorylation and aggregation. Although oxidative stress has been reported to alter the expression and post-translational modification of SELENOW^39,40^, we did not observe any effect of SELENOW overexpression on the oxidative stress marker MDA in our AD model. This was consistent with our previous report that SELENOW KO did not alter the MDA levels in the cortex^31^. Yet, we detected relevant changes in glial cells in the hippocampus of SELENOW-AD mice. These changes were primarily characterized by a reduction in the microglial marker Iba-1, which is associated with neuroinflammation, and an increase in the oligodendrocyte myelination marker Oligo2. These improvements in AD by SELENOW may be attributed to the amelioration of tau-related pathology. Therefore, our subsequent discussion will primarily focus on SELENOW and tau.

A former study identified that SELENOW Cys37 and tau Cys322 form a disulfide bond and inhibit in vitro tau aggregation^15^, but the relationship between SELENOW and tau has not been established yet. Our data suggested that SELENOW expression was inversely correlated with tau levels in AD, indicating its role in regulating tau homeostasis. The regulatory capacity of SELENOW on tau expression appears to stem from its disulfide bond interaction with tau, rather than through the selenocysteine containing TXN-like motif. This is supported by our observation that another cytoplasmic selenoprotein, SELENOV, possessing a comparable TXN-like motif, exhibited no discernible impact on tau expression levels. The protein half-life study indicated that SELENOW promotes the degradation of tau. By applying different degradation pathway inhibitors, we confirmed that SELENOW mediated tau degradation through the UPS, similar to cochaperone proteins like BAG2, DnaJA1 and Hop^26,41,42^. The cellular clearance machinery of tau has been promoted while upregulating these cochaperones of Hsp70 and Hsp90^43^, suggesting that SELENOW may affect the Hsp70/Hsp90 machinery, leading to tau degradation. Hsp70 forms a complex with tau to prevent aggregate formation and enhance stability^44^. Our findings revealed that upregulation of Hsp70 counteracted the effects of SELENOW on tau. The IP and PLA results revealed that SELENOW may compete with Hsp70 for interaction with tau. Hsp70 recognized specific motifs in tau located near the SELENOW binding site Cys322 in the MTBR^45^. We proposed that SELENOW might promote tau clearance by interference with the Hsp70-tau complex.

Studies have highlighted the importance of tau PTMs in pathological conditions and tau degradation^46^. A noticeable point was that tau acetylation at lysine residues promotes Hsp70 recruitment^47^ while the Cys322 site was crucial for tau auto-acetylation. Our data showed that tau K281 acetylation was inhibited in the case of SELENOW overexpression. For UPS degradation, ubiquitin labels are also generally attached to lysine residues^48^. Correspondingly, we detected increased ubiquitination levels of tau in the presence of SELENOW. Tau K281 acetylation may accelerate AD progression by disrupting MT stabilization, enhancing fibrillar tau aggregate formation, and disturbing mitochondrial homeostasis^49,50^. SELENOW may relieve tau-related problems by blocking the Cys322 site, reducing auto-acetylation of tau at K281, decreasing the Hsp70-tau complex, and promoting poly-ubiquitination and UPS degradation of tau.

SELENOW is extensively expressed in axonal and synaptic sites. In mice lacking SELENOP, the selenoprotein that is responsible for selenium brain transportation, SELENOW has been found to be significantly decreased in synaptosomes^13^. Although our experiments in cell models revealed that upregulating SELENOW could reduce the expression levels of Tau, we did not observe a corresponding tau protein overexpression upon SELENOW knockout. Instead, SELENOW KO resulted in a decrease in hippocampus tau protein expression, accompanied by a notable loss in neuronal processes and synapses. Physiologically, tau is enriched in the axon but is also localized to synapses^51^. We speculated that SELENOW, together with tau, might be involved in the maintaining of structural and functional of neuron processes and terminals. The knockout of SELENOW affected synaptic formation, leading to a corresponding decrease in tau protein levels. This was further supported by similar phenotypes that we observed in SELENOW KO mice and that were reported in *Mapt* + */−* (tau knockout) mice, including increased locomotor activity and impaired LTP. Both tau and SELENOW interact with the 14-3-3 protein^40,52^, suggesting they may function together under normal conditions.

Notably, we found that SELENOW reduced tau specifically in the 4-repeat isoform in our mouse model. Our aforementioned ICP‒MS/MS results also highlighted the effect of SELENOW on the PTMs of peptides from the R2 repeat, which was unique to the 4-repeat isoform. These results indicated an isoform preference of SELENOW in altering tau homeostasis. Interestingly, Hsp70 has been shown to be more effective at inhibiting the aggregation of the 3-repeat tau isoforms^23^. As predicted by ComplexContact (Supplementary Fig. 6), it is possible that the 4-repeat isoform of tau is more inclined to interact with SELENOW instead of forming a complex with Hsp70 due to an extra cysteine residue in the MTBR. Nevertheless, more studies are needed to elucidate the underlying mechanism.

In conclusion, as shown in Fig. 8c, our study found that SELENOW protein levels were inversely correlated with tau, and restoring SELENOW levels showed beneficial effects by mitigating tau-related symptoms in an AD mouse model. SELENOW competed with Hsp70 for tau interaction, facilitating its degradation through the UPS. SELENOW knockout led to synaptic defects, reduced synaptic vesicle density, tau dysregulation and impaired cognitive functions in the mouse hippocampus. Considering selenium’s potential in preventing and treating AD, along with the upregulation of SELENOW by selenium levels, our findings suggest that SELENOW could be considered as a potential target of selenium for modulating tau homeostasis in AD. However, further research is necessary to fully elucidate its therapeutic efficacy across various AD models and potentially in clinical trials.

## Methods

### Animals and ethics statement

Triple-transgenic AD (Stock No.004807, 3×Tg AD) mice homozygous for three AD-related mutant alleles (Psen1 M146V mutation, APPSwe and tauP301L) and background control mice (B6129SF2/J) were purchased from Jackson Laboratory. Homozygous SELENOW (Gene ID: 20364) knockout (referred to as SELENOW KO below) mice and WT mice (C57BL/6 N) used as background controls were generated and bred by BIOCYTOGEN (Beijing, China). The mice were housed and bred in cages under standard conditions with a 12:12 h light-dark cycle and 22 °C room temperature. The use of animals was approved by the Ethics Committee of Shenzhen University. All animal experiments were performed in accordance with the guidelines of the Institutional Animal Care and Use Committee of the Institute for Nutritional Sciences in China. We have complied with all relevant ethical regulations for animal use.

### Cell lines, plasmids, and siRNA interference

HEK293 and HEK293 cells that were stably transfected with flag-tagged full-length tau protein (HEK293TAU) were maintained in DMEM supplemented with 10% fetal bovine serum and 100 U/ml penicillin/streptomycin in a 5% CO_2_ 37 °C incubator. HEK293 cells were used as a negative control in our experiments. Specific complex machinery, such as SECIS elements, is required to cotranslationally incorporate selenocysteine (Sec, U) into selenoproteins, and it is encoded by UGA, which is recognized as a stop codon in general cases. Thus, all the U residues were substituted with cysteine (Cys, C), which showed similar chemical properties, for efficient overexpression of SELENOW and Selenoprotein V (SELENOV) in our study. Substitution of SELENOW C37 with serine, the C37S mutant, was also generated to study the binding site between SELENOW and tau. Full-length human SELENOW (NM_003009), and SELENOV (NM_182704.2) with corresponding point mutations was cloned into the pCDNA3.1 vector for overexpression in cell lines, and full-length human SELENOW with the U13 residue mutated to C was cloned into the AAV vector pAV-hSyn-P2A-GFP for overexpression in mouse brains. Specific siRNAs for SELENOW (sc-40932) and their scramble control (sc-37007) were purchased from Santa Cruz Biotechnology. The siRNAs were transfected into cells to interfere with SELENOW expression. TransEasy transfection reagent (FOREGENE) was used to transfect the constructs or siRNAs into cells. Cells were grown 48−72 h after transfection.

### Chemicals and antibodies

Chemicals including CHX (HY-12320), MG-132 (HY-13259), CQ (HY-17589) and TRC051384 (HY-101712) were purchased from MedChemExpress (MCE). Sodium selenate (S8295) was purchased from Sigma. CHX is a eukaryote protein synthesis inhibitor. To study the half-life of tau protein, 50 μg/mL CHX was applied to cells for a time gradient from 0 to 12 h. MG-132 and CQ are inhibitors of the 20 S proteasome and autophagy, respectively, while TRC051384 is an inducer of heat shock proteins. In our experiments, 10 μM MG-132, 20 μM CQ or 10 μM TRC051384 was applied to cells for 6 h, and 10 or 100 μM selenate was applied to cells for 24 h.

Two different anti-tau protein antibodies, tau5 from Abcam (ab80579, monoclonal) and HT7 from Invitrogen (MN1000, monoclonal), were used in our experiments. For detection of tau phosphorylation at different epitopes, the anti-pTau181 from Abcam (ab75679, polyclonal), anti-pTau 202/205 from Abclonal (AP1378, polyclonal), anti-pTau231 from Abcam (ab151559, monoclonal), anti-pTau262 from Abclonal (AP0397, polyclonal), anti-pTau396 from Abcam (ab109390, monoclonal), anti-pTau404 from Abclonal (AP1378, monoclonal), anti-pTau416 from Abclonal (AP1101, polyclonal) and anti-pTau 422 from Abcam (ab79415, monoclonal) were used in our experiments. For detection of different tau isoforms, the anti-Tau 4-repeat isoform RD4(# 05-804, monoclonal) and anti-Tau 3-repeat isoform RD3(#05-803, monoclonal) were purchased from Merck Millipore. The anti-SELENOW antibody was purchased from Rockland Immunochemical (600-401-A29, polyclonal). Anti-myc (2276, monoclonal), anti-GFP (2956, monoclonal), anti-Hsp70 (4872, polyclonal), anti-LC3A/B (12741, monoclonal), anti-ubiquitin (3936, monoclonal), and anti-acetylated lysine (9441, polyclonal) antibodies were purchased from Cell Signaling Technology (CST). For detection of oxidative stress, synaptic and glia protein markers, the anti- malondialdehyde (MDA, ab27642, polyclonal), anti-synaptophysin (ab32127, monoclonal) and anti-PSD95 (ab18258, polyclonal) were purchased from Abcam, anti-Iba1(17198, monoclonal) was purchased from CST, anti-Oligo2 (66513-1-lg, monoclonal) and anti-GFAP (16528-1-AP, polyclonal) were from Proteintech. Anti-GAPDH antibody was from ABclonal (A19056, monoclonal). Alexa Fluor 555-conjugated anti-mouse secondary antibodies and DAPI staining solution were obtained from CST. Peroxidase-conjugated anti-mouse/rabbit antibodies were from Abmart (m21001, m21002).

### Proximity Ligation Assay (PLA)

Whole sets of Duolink® reagents for PLA fluorescence detection were purchased from Sigma‒Aldrich, and the experiments were carried out according to the manufacturer’s instructions. Generally, HEK293TAU cells cultured on coverslips were transfected with SELENOW(U13C) GFP plasmids. Forty-eight hours after transfection, the cells were fixed in 4% polyformaldehyde (Sigma) at 4 °C for 30 min and permeabilized with 0.1% Triton X-100 in TBS at room temperature for 15 min. The coverslips were then blocked with blocking solution at 37 °C for 60 min and incubated with properly diluted primary antibodies (anti-tau5 and anti-Hsp70, anti-tau5 and anti-ubiquitin) at 4 °C overnight. Then, they were incubated with PLUS and MINUS PLA probes at a dilution of 1:5 for 60 min at 37 °C. The ligation and amplification steps were performed at 37 °C by incubation with the corresponding ligase or polymerase. After final washes, the coverslips were mounted with mounting media with DAPI and imaged under an OLYMPUS BX53 microscope.

### Quantitative Real-Time PCR (Q-PCR)

Q-PCR was employed to detect the gene expression of MAPT and HSPs in response to different treatments. A kit was used to extract total RNA from cells according to the manufacturer’s instructions (Fastagen, RNAfast200). The RNA templates were quantified by Nanodrop (Thermo) and reverse transcribed to cDNA using a reverse transcription kit with gDNA eraser (Takara, RR047A). qPCRs were performed on the QuantStudio real-time PCR (ABI) with SYBR Color qPCR master mix (Vazyme, Q441-02). The following thermal cycling conditions were used: 5 min at 95 °C, followed by 40 cycles of 95 °C for 15 s and 60 °C for 1 min. The 2^−ΔΔCT^ method was applied to calculate the relative gene expression. ACTB was chosen as an endogenous reference gene to normalize the Q-PCR data. The sequences of primer pairs for Q-PCR are listed in Supplementary Table 1.

### Western blotting and coimmunoprecipitation

Whole cell or tissue extracts were prepared by lysing in commercial WB and IP lysis buffer from Beyotime (P0013, with protease inhibitor cocktail) on ice for 30 min and then ultrasonication. The lysates were spun at 20,000 × g for 15 min at 4 °C. The supernatants were then used in western blotting experiments. BCA protein assay kits (Thermo) were used to measure the protein concentration, and equal amounts of total proteins were loaded onto SDS‒PAGE gels. Proteins were transferred to a PVDF membrane at 100 V for 90 min. Blots were blocked in TBST with 5% nonfat dried milk for 2 h and incubated with primary antibody at the appropriate concentration at 4 °C overnight. Blots were then rinsed 3 × 15 min in TBST and incubated with peroxidase-conjugated secondary antibody (1:5000) for 2 h at room temperature.

For coimmunoprecipitation (co-IP), 4 μg antibodies or IgG were mixed with 50 μl Protein A/G magnetic beads (MCE) for 2 h at 4 °C, washed 3 × 5 min with IP buffer, and then incubated with 600 μg total protein from lysis at 4 °C overnight. After washing 3 × 5 min with IP buffer, immunoprecipitates were analyzed by immunoblotting.

### LC‒MS/MS detection of tau protein posttranslational modification

The tau proteins for liquid chromatography–tandem mass spectrometry (LC‒MS/MS) were immunoprecipitated from whole cell extracts of HEK293TAU cells that were transiently transfected with SELENOW or its C37S mutant. Generally, as described by ref. ^53^. the immunoprecipitated proteins were excised from Coomassie blue-stained SDS‒PAGE gels and trypsinized, and the resulting peptides were subjected to LC‒MS/MS on a hybrid linear ion trap-Orbitrap mass spectrometer (LTQ-Orbitrap, Thermo Scientific) by Wininnovate Bio (Shenzhen, China). Mass spectra were searched against full-length human tau (441 aa) using the search engine PEAKS. The search parameters included 10.0 ppm peptide mass tolerance, 0.05 Da fragment tolerance, fixed modification carbamidomethylation: +57.02 and variable modifications oxidation: +15.99, acetylation: +42.01, phosphorylation: +79.97, deamidation: +0.98, Pyro-glu from Q: −17.03 and Pyro-glu from E: −18.01. The resulting filtration parameters were set as peptide −10lgP ≥ 0, peptide Ascore ≥ 0, protein −10lgP ≥ 20, unique peptides ≥ 0, and de novo ALC score ≥ 50%.

### Electrophysiology

Six-month-old WT and SELENOW KO mice were anesthetized with isoflurane and decapitated. Their brains were quickly isolated and placed into ice-cold artificial cerebrospinal fluid (ACSF) continuously bubbled with carbogen (95% O_2_/5% CO_2_) containing 119 mM sucrose, 2.5 mM KCl, 2.5 mM CaCl_2_·2H_2_O, 26.2 mM NaHCO_3_, 1.0 mM NaH_2_PO_4_·2H_2_O, 1.3 mM MgCl_2_·6H_2_O, and 11.0 mM glucose and cut into 300 mM slices on a vibrating blade microtome (Leica VT1200). Slices were transferred to a holding chamber at 32 °C for 2 h in ACSF with carbogen. Field excitatory postsynaptic potential (fEPSP) values were recorded using an MED64 multichannel recording system (array size: 1 × 1 mm; each electrode: 50 × 50 μm; interpolar distance: 150 μm; electrode array: 8 × 8; Alpha Med Systems, Osaka, Japan). For each slice, the baseline stimulus intensity was set at −10 μA every 20 s for 30 min. LTP was induced with a conditioning stimulus consisting of three theta burst trains (ten 5-Hz series of four 100-Hz pulses each, 40 s apart). At least eight slices from six mice were used in each group.

### AAV9 brain injection

Six-month-old WT and 3×Tg-AD mice were used for adeno-associated virus (AAV) brain injection. The AAV-empty vector pAV-hSyn-P2A-GFP with a specific neuronal promoter (synpasin-1) and GFP label and the pAV-hSyn-SELENOW(U13C)-P2A-GFP clone were constructed and AAV9-packaged by WZ Biosciences (Shandong, China). Then, the injection procedures were performed. Briefly, the mice were anesthetized by isoflurane administration through a modified face mask, and their heads were properly secured in a stereotaxic device. With a 10 μl Hamilton syringe, 2 μl (3.09 × 10^13^ vg/ml) AAV9 was bilaterally injected into the hippocampal CA3 region (X = ± 2.30 mm, Y = −2.18 mm, Z = −2.10 mm) of the mouse brain. Animals were then followed until they were 12 months old, when they underwent behavioral tests, and two weeks later euthanized for brain tissue staining and western blotting.

### Behavioral tests

Behavioral tests, including the open field test (OFT), elevated plus maze, Y-maze, novel object recognition, contextual fear conditioning and Morris water maze tests, were applied as we previously reported^31^ to evaluate the anxiety degree, locomotor activity, spatial learning ability and memory capacity of mice.

Generally, the OFT tasks were performed in a L100 × W100 × H30 cm open-top chamber in a quiet room. The bottom of the chamber was divided into 25 squares with a size of L20 × W20 cm. The mice were placed in the center of the chamber and allowed to explore the open field for 5 min. For each mouse, the numbers of grid crossings, rearing, and defecation were recorded. After each trial, the mice were then removed from the chamber and returned to their home cages, and the chamber was cleaned with 70% ethanol.

Briefly, the elevated plus maze apparatus was a cross-shaped maze that consisted of four arms with an L30 × W5 × H60 cm. Two closed arms were equipped with a 30 cm high wall, while two open arms were not. The mouse was initially placed facing an open arm in the center of the maze and then allowed to explore the maze for 5 min. Mice that fell off the maze were excluded. The time each mouse spent in the open and closed arms was recorded.

The Y-maze tests were performed in a Y-shaped maze with three identical arms at a 120° angle from each other. The mice were placed in the center of the maze and were given free access to all three arms during a session lasting 5 min. If the mice chose a new arm than the one it visited previously, it was defined as an alteration. The total number of arm entries and the sequence of entries were manually recorded, and then the spontaneous alternation ratio and the number of total entries were analyzed. The spontaneous alternation ratio was defined as N_alt_/(N_total_-2). [N_alt_: number of alternations, an alternation is achieved when an animal enters a new arm rather than returning to one visited previously. N_total_: number of total arm entries]. After each trial, the mice were then returned to their home cages, and the maze was cleaned with 70% ethanol.

The novel object recognition tasks were conducted in an L40 × W40 × H40 cm open-top square chamber in the test room equipped with a daylight lamp. This box was divided into 25 equal squares. In the habituation phase, each mouse was placed in the empty box for 5 min to habituate it to the environment and to the apparatus. The test sessions were performed the next day. In the acclimation phase, home cages were left for acclimation in the test room for 1 h prior to the beginning of the test. In the acquisition phase, each mouse was placed for 5 min in the box containing two identical objects. After a 90 min retention interval back to its home cage, the mouse was then placed into the box and exposed to one of the familiar objects and to a novel object for a short-term recognition memory test. The mouse was initially placed in the apparatus facing the wall and allowed to explore the objects for 5 min. When the mouse’s nose pointed or touched an object within 1 cm, it was recorded as an exploration behavior. The number of times the mouse explored one of the two objects was recorded. The discrimination ratio was defined as (T_new_− T_old_)/T_total_; T_new_: exploration times for the new object, T_old_: exploration times for the old object, and T_total_: total exploration times.

The contextual fear conditioning experiments were conducted in two enclosed chambers from Sansbio (Jiangsu, China) equipped with top-view infrared cameras, decibel meters, controllable loudspeakers, white lights, and electric barriers. The freezing behavior of the mice was analyzed using tracking software. On the first day (training stage), mice were placed in the chamber and recorded for 2 min as a baseline, and then they were trained with four pairs of tone (CS) and an electric foot shock (US). One pair of CS-US consisted of 30 s of tone (6000 Hz, 80 dB) followed by 2 s electric foot shock (0.35 mA). On the next day (testing stage), to test their contextual fear memory retention, the mice were re-exposed to the same conditioned chamber for 5 min without giving any tone and foot shock. One hour later, the mice were tested for novel condition fear memory and tune fear memory. The mice were exposed to an altered context with a different chamber shape and olfactory cues for 3 min as the novel condition fear memory. Then, the mice were given 30 s of tone (6000 Hz, 80 dB) as auditory cues, and another 3 min was recorded as the tune fear memory. The percent freezing was defined as freezing time/total time.

The Morris water maze tasks were performed in a water-filled round pool with a size of D120 × H50 cm. The temperature of the water was kept at 22 ± 1 °C, and the depth of the water was 26 cm. A camera was mounted on the top of the pool to track the movement of the mice. The pool was divided into four quadrants. A round plastic escape platform with a diameter of 12 cm was placed in one of the quadrants, with its surface 1−2 cm below the water level. Each mouse was manually guided to the platform for 15 s at first and then placed in the opposite quadrant. The time for the mouse to seek the platform was recorded by the tracking system within 120 s as the escape latency. The training trials were performed for five consecutive days. Then, after 24 h and 72 h, the platform was removed. In addition, 120-s probe trials were conducted to assess short- and long-term memory. The time for the mouse to spend in the target quadrant and the number of times the mouse crossed the platform were recorded and analyzed by SMART v3.0 (Panlab) software.

### Brain tissue staining and imaging

Freshly separated mouse brains were fixed in 4% polyformaldehyde (Sigma) at 4 °C overnight. Then, the brain tissues were dehydrated, embedded in paraffin wax or OCT, and cut into 10 μm slides for staining. After staining, tissue slides were mounted with antifade mounting medium and imaged under an OLYMPUS BX53 microscope.

For immunostaining, slides were permeabilized with 0.1% Triton X-100 in TBS and blocked in 3% BSA in TBS containing 0.1% Triton X-100 for 1 h at room temperature. Then, they were incubated overnight at 4 °C with primary antibody at an optimal dilution. The coverslips were washed 3 × 5 min with TBS containing 0.1% Tween 20 and incubated with Alexa Fluor 555-conjugated secondary antibodies at a dilution of 1:500 for 1 h at room temperature. DAPI staining was performed at a dilution of 1:1000 for 5 min at room temperature.

For Bielschowsky silver staining of NFTs, commercial staining kits (HPBIO-JM4573) from HEPENGBIO were used. Frozen sections of mouse brain slides were fixed and stained following the manufacturer’s instructions. For transmission electron microscopy, the hippocampus isolated from the mouse brain was fixed with 2.5% glutaraldehyde and 1% osmium tetroxide for 2 h at 4 °C, dehydrated in a graded ethanol series, and embedded in epoxy resin. Then, samples were sectioned into ultrathin slices (DiATOME knife and Leica ultramicrotome) and stained with 2% uranyl acetate and 2% lead citrate. Images were acquired using an FEI Tecnai G2 Spirit transmission electron microscope by Servicebio (Wuhan, China).

### Statistics and reproducibility

All the data are representative of at least three independent experiments. Data are presented as the mean ± SD. Statistical comparisons were performed using *t* tests between two groups, post-hoc Tukey multiple comparisons were performed after one-way ANOVA for groups that had more than two, and post-hoc Sidak multiple comparisons tests were performed after two-way ANOVA for groups that had more than two and two categorical independent variables. Outliers were detected and excluded using the Grubbs’ test. *p < 0.05, **p < 0.01 and ***p < 0.001 were considered to be statistically significant.

### Reporting summary

Further information on research design is available in the Nature Portfolio Reporting Summary linked to this article.

### Supplementary information

Supplementary Information

Description of Additional Supplementary Files

Supplementary Data

reporting summary

## Data Availability

The data that support the findings of this study are available in the Supplementary Data file, and the uncropped blots are provided as Supplementary Fig. 7. All other data are available from the corresponding author on reasonable request.

## References

[1] Young ZT, Mok SA, Gestwicki JE (2018). Therapeutic strategies for restoring Tau homeostasis. Cold Spring Harb. Perspect. Med..

[2] Chen J, Berry MJ (2003). Selenium and selenoproteins in the brain and brain diseases. J. Neurochem..

[3] Varikasuvu SR, Prasad VS, Kothapalli J, Manne M (2019). Brain Selenium in Alzheimer’s Disease (BRAIN SEAD Study): a systematic review and meta-analysis. Biol. Trace Elem. Res..

[4] Nascimento CQD (2021). Selenium concentrations in elderly people with Alzheimer’s disease: a cross-sectional study with control group. Rev. Brasileira de. Enferm..

[5] R. Cardoso B (2017). The APOE ε4 allele is associated with lower selenium levels in the brain: implications for Alzheimer’s Disease. ACS Chem. Neurosci..

[6] Van der Jeugd A (2018). Reversal of memory and neuropsychiatric symptoms and reduced tau pathology by selenium in 3xTg-AD mice. Sci. Rep..

[7] Zhang ZH (2017). Long-term dietary supplementation with selenium-enriched yeast improves cognitive impairment, reverses synaptic deficits, and mitigates Tau Pathology in a triple transgenic mouse model of Alzheimer’s Disease. J. Agric. Food Chem..

[8] Zhang ZH (2017). Selenomethionine mitigates cognitive decline by targeting both Tau hyperphosphorylation and autophagic clearance in an Alzheimer’s disease mouse model. J. Neurosci..

[9] van Eersel J (2010). Sodium selenate mitigates tau pathology, neurodegeneration, and functional deficits in Alzheimer’s disease models. Proc. Natl Acad. Sci. USA.

[10] Whanger PD (2000). Selenoprotein W: a review. Cell. Mol. Life Sci..

[11] Zhang Y (2008). Comparative analysis of selenocysteine machinery and selenoproteome gene expression in mouse brain identifies neurons as key functional sites of selenium in mammals. J. Biol. Chem..

[12] Sasuclark AR, Khadka VS, Pitts MW (2019). Cell-type specific analysis of selenium-related genes in brain. Antioxidants.

[13] Raman AV (2013). Selenoprotein W expression and regulation in mouse brain and neurons. Brain Behav..

[14] Yeh JY (1997). Dietary selenium increases selenoprotein W levels in rat tissues. J. Nutr..

[15] Chen H (2018). Blocking the Thiol at Cysteine-322 destabilizes Tau protein and prevents its oligomer formation. ACS Chem. Neurosci..

[16] Lee MJ, Lee JH, Rubinsztein DC (2013). Tau degradation: the ubiquitin-proteasome system versus the autophagy-lysosome system. Prog. Neurobiol..

[17] Alkan Z, Duong FL, Hawkes WC (2015). Selenoprotein W controls epidermal growth factor receptor surface expression, activation and degradation via receptor ubiquitination. Biochim. et. Biophys. Acta.

[18] Nettleford, S. K. et al. Selenoprotein W Ameliorates experimental colitis and promotes intestinal epithelial repair. Antioxidants. **12**, 850 (2023). 10.3390/antiox12040850 PMC10134982 37107231

[19] Xu M (2018). A systematic integrated analysis of brain expression profiles reveals YAP1 and other prioritized hub genes as important upstream regulators in Alzheimer’s disease. Alzheimer’s Dement..

[20] Kao SH (2015). Analysis of protein stability by the cycloheximide chase assay. Bio-Protoc..

[21] Gorantla NV, Chinnathambi S (2018). Tau protein squired by molecular chaperones during Alzheimer’s disease. J. Mol. Neurosci..

[22] Young ZT (2016). Stabilizing the Hsp70-Tau complex promotes turnover in models of tauopathy. Cell Chem. Biol..

[23] Voss K, Combs B, Patterson KR, Binder LI, Gamblin TC (2012). Hsp70 alters tau function and aggregation in an isoform specific manner. Biochemistry.

[24] Moll A (2022). Hsp multichaperone complex buffers pathologically modified Tau. Nat. Commun..

[25] Zeng H (2018). ComplexContact: a web server for inter-protein contact prediction using deep learning. Nucleic Acids Res..

[26] Abisambra JF (2012). DnaJA1 antagonizes constitutive Hsp70-mediated stabilization of tau. J. Mol. Biol..

[27] Cohen TJ, Friedmann D, Hwang AW, Marmorstein R, Lee VM (2013). The microtubule-associated tau protein has intrinsic acetyltransferase activity. Nat. Struct. Mol. Biol..

[28] Kontaxi C, Piccardo P, Gill AC (2017). Lysine-directed post-translational modifications of Tau Protein in Alzheimer’s disease and related Tauopathies. Front. Mol. Biosci..

[29] Kimura T (2014). Microtubule-associated protein tau is essential for long-term depression in the hippocampus. Philos. Trans. R. Soc. Lond. Ser. B, Biol. Sci..

[30] Biundo F, Del Prete D, Zhang H, Arancio O, D’Adamio L (2018). A role for tau in learning, memory and synaptic plasticity. Sci. Rep..

[31] Situ J (2022). Comparative proteomic analysis reveals the effect of Selenoprotein W deficiency on oligodendrogenesis in fear memory. Antioxidants.

[32] Zhang ZH (2021). Selenium restores synaptic deficits by modulating NMDA receptors and Selenoprotein K in an Alzheimer’s disease model. Antioxid. Redox Signal..

[33] Aaseth J (2016). Treatment strategies in Alzheimer’s disease: a review with focus on selenium supplementation. Biometals.

[34] Zhang ZH, Song GL (2021). Roles of Selenoproteins in brain function and the potential mechanism of Selenium in Alzheimer’s Disease. Front. Neurosci..

[35] Fagerberg L (2014). Analysis of the human tissue-specific expression by genome-wide integration of transcriptomics and antibody-based proteomics. Mol. Cell. Proteom..

[36] Kim H (2021). Selenoprotein W ensures physiological bone remodeling by preventing hyperactivity of osteoclasts. Nat. Commun..

[37] Misra S (2023). Loss of selenoprotein W in murine macrophages alters the hierarchy of selenoprotein expression, redox tone, and mitochondrial functions during inflammation. Redox Biol..

[38] Miao Z, Wang W, Miao Z, Cao Q, Xu S (2024). Role of Selenoprotein W in participating in the progression of non-alcoholic fatty liver disease. Redox Biol..

[39] Ko KY (2019). S-Glutathionylation of mouse selenoprotein W prevents oxidative stress-induced cell death by blocking the formation of an intramolecular disulfide bond. Free Radic. Biol. Med..

[40] Jeon YH (2016). Identification of a redox-modulatory interaction between selenoprotein W and 14-3-3 protein. Biochim. et. Biophys. Acta.

[41] Carrettiero DC, Hernandez I, Neveu P, Papagiannakopoulos T, Kosik KS (2009). The cochaperone BAG2 sweeps paired helical filament- insoluble tau from the microtubule. J. Neurosci..

[42] Jinwal UK, Koren J, Dickey CA (2013). Reconstructing the Hsp90/Tau machine. Curr. Enzym. Inhibit..

[43] Rutledge BS, Choy WY, Duennwald ML (2022). Folding or holding?-Hsp70 and Hsp90 chaperoning of misfolded proteins in neurodegenerative disease. J. Biol. Chem..

[44] Kundel F (2018). Hsp70 inhibits the nucleation and elongation of Tau and Sequesters Tau aggregates with high affinity. ACS Chem. Biol..

[45] Jinwal UK (2013). Imbalance of Hsp70 family variants fosters tau accumulation. FASEB J..

[46] Wang Y, Mandelkow E (2016). Tau in physiology and pathology. Nat. Rev. Neurosci..

[47] Choi H (2020). Acetylation changes tau interactome to degrade tau in Alzheimer’s disease animal and organoid models. Aging Cell.

[48] Sadowski M, Sarcevic B (2010). Mechanisms of mono- and poly-ubiquitination: ubiquitination specificity depends on compatibility between the E2 catalytic core and amino acid residues proximal to the lysine. Cell Div..

[49] Liu Q (2023). Acetylated tau exacerbates learning and memory impairment by disturbing with mitochondrial homeostasis. Redox Biol..

[50] Trzeciakiewicz H (2017). A dual pathogenic mechanism links tau acetylation to sporadic tauopathy. Sci. Rep..

[51] Hanger DP, Goniotaki D, Noble W (2019). Synaptic Localisation of Tau. Adv. Exp. Med. Biol..

[52] Chen Y (2019). 14-3-3/Tau interaction and tau amyloidogenesis. J. Mol. Neurosci..

[53] Huseby CJ (2019). Quantification of Tau Protein Lysine Methylation in Aging and Alzheimer’s Disease. J. Alzheimer’s Dis..

