# Supplementary material for: Selenoprotein W modulates tau homeostasis in an Alzheimer’s disease mouse model
Source: Commun Biol. 2024 Jul 17;7:872. doi: 10.1038/s42003-024-06572-0 (PMC11255228; doi:10.1038/s42003-024-06572-0)
Supplement: Supplementary file 1 — Supplementary Information [file 42003_2024_6572_MOESM1_ESM.pdf]

Supplementary Materials

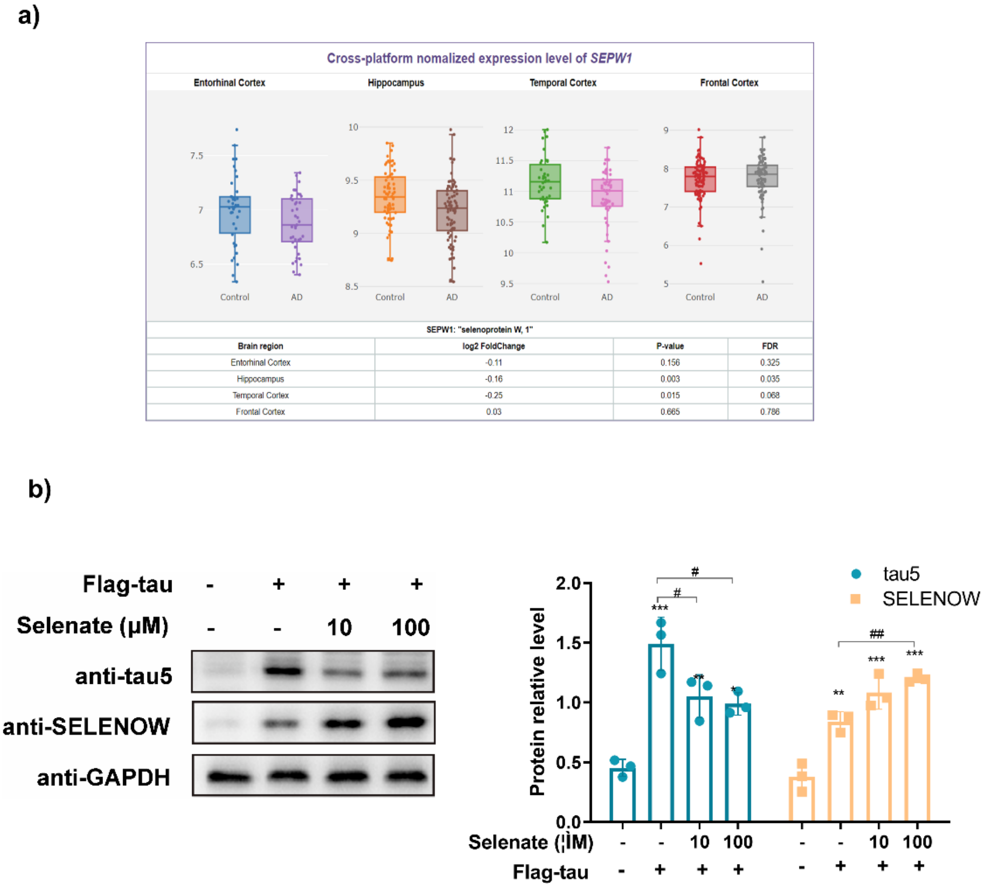

Supplementary Figure 1.

a) SEPW1 gene expression profiling in control and AD brains provided by Alzdata. The differential expression of SEPW1 in four brain regions, including the entorhinal cortex, hippocampus, temporal cortex and frontal cortex, is represented as the log2-fold change. The P value and false discovery rate (FDR) were measures of the statistical significance of the expression difference.

b) HEK293TAU cells were treated with or without selenate for 24 h. Representative western blot showed that selenium supplementation by 10 and 100 µM selenate induced the upregulation of SELENOW and reduced the expression of tau.

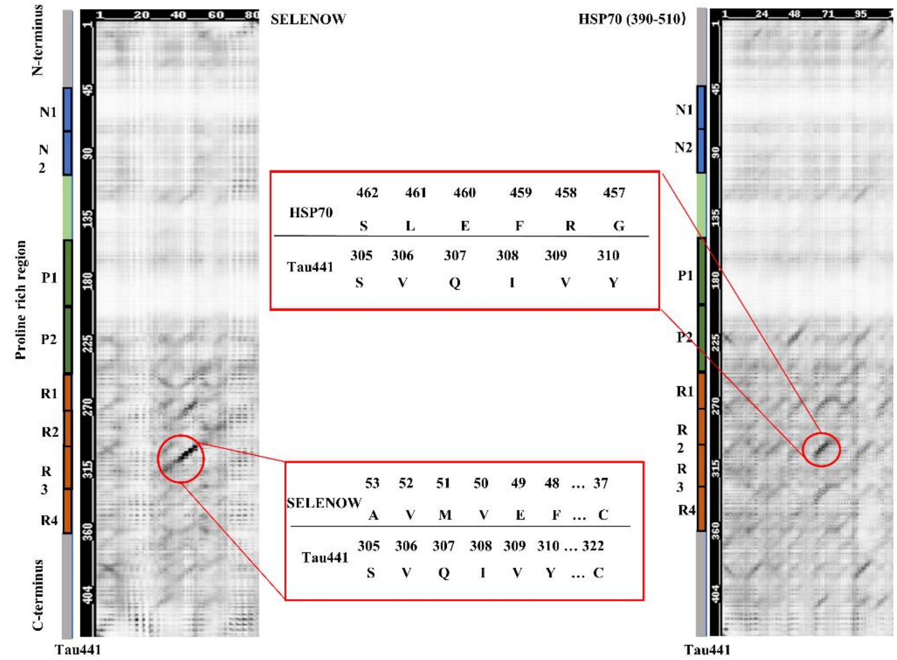

Supplementary Figure 2.

The ComplexContact predicted interfacial contacts between SELENOW and full length Tau441 (left panel), and between Hsp70 substrate binding domain (390-510) and full length Tau441 (right panel). The amino acid sequences within the interfacial contacts were provided in the red boxes.

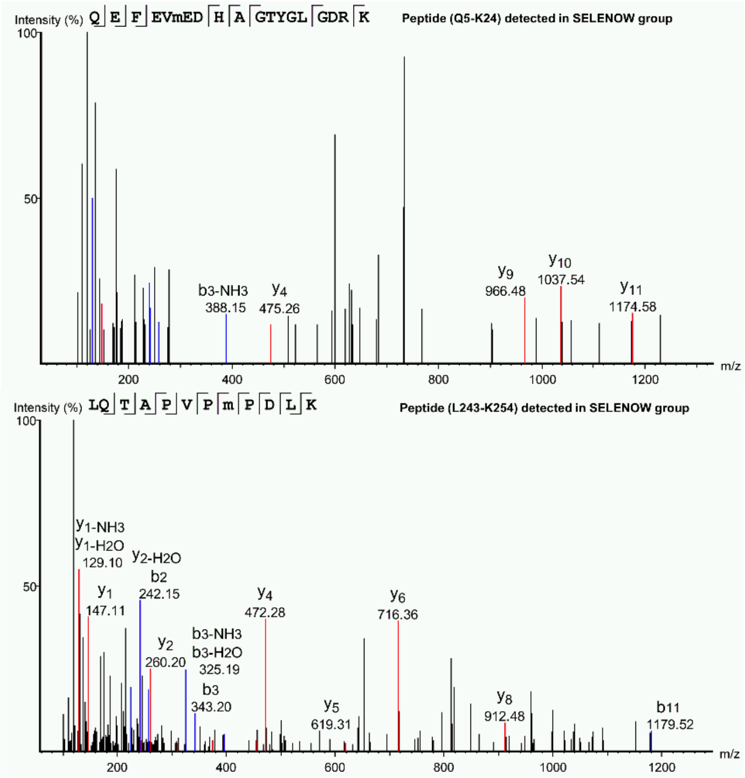

Supplementary Figure 3. Peptide fragments from Q6 to K24 and L243 to K254 detected by mass spectrometric analysis in SELENOW group, which revealed oxidation at M11 and M250.

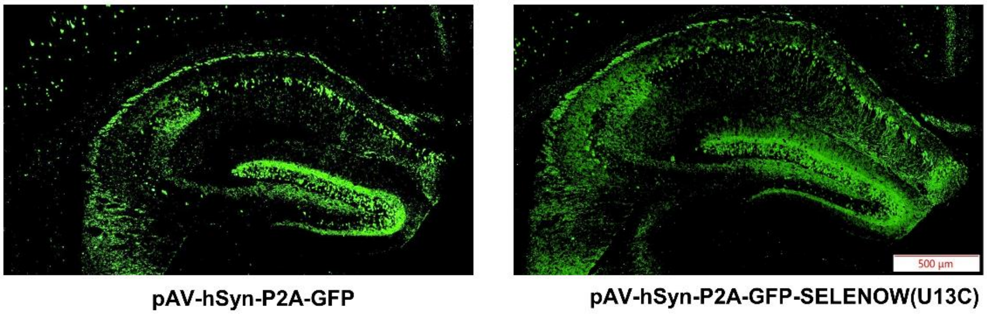

Supplementary Figure 4. Representative micrographs of hippocampus from the AAV-injected mouse brains showing the overall expression of GFP. Scale bar = 500μm.

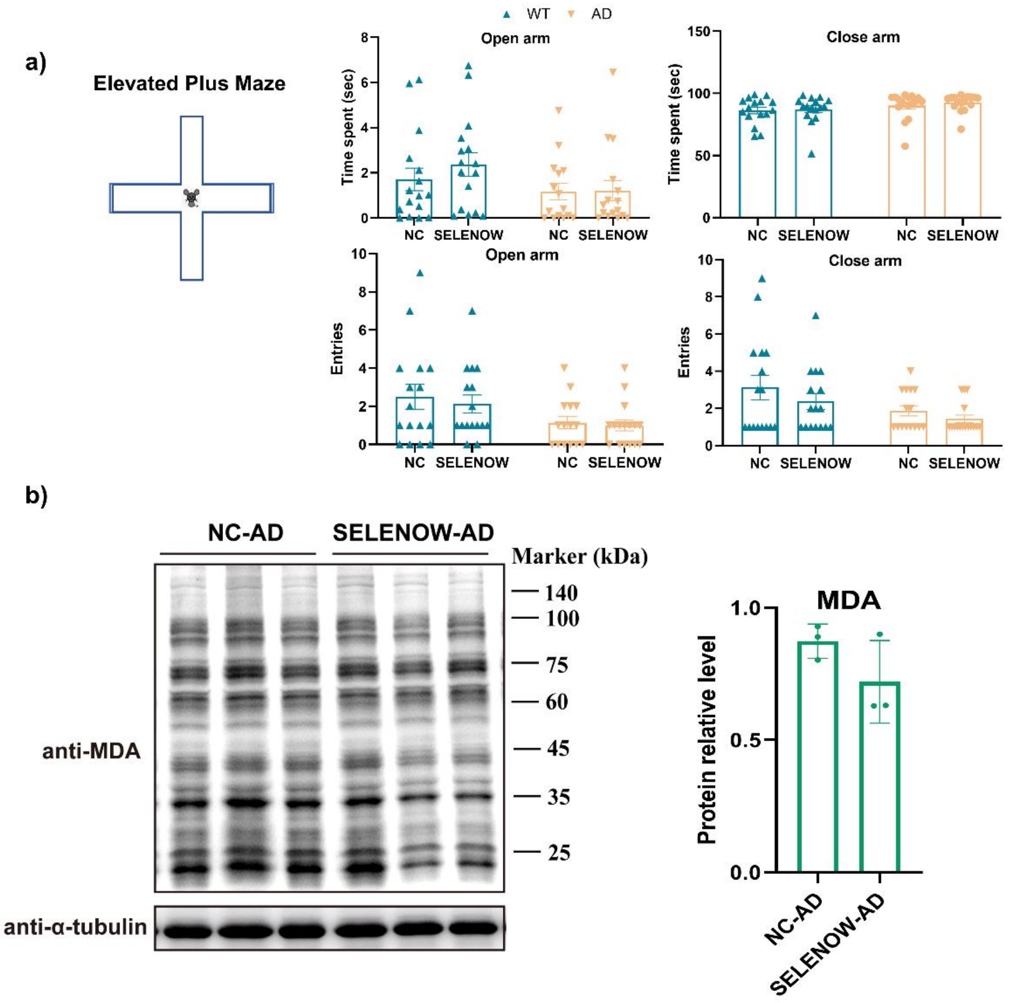

Supplementary Figure 5.

a) Anxiety in 12-month-old NC-AD and SELENOW-AD mice was evaluated by the time spent in the open arms and closed arms of the elevated plus maze test.

b) Representative western blot results and analysis of Malondialdehyde (MDA) in brain hippocampus homogenates from 12-month-old 3×Tg AD mice receiving empty vector (NC-AD) or AAV-SELENOW (SELENOW-AD).

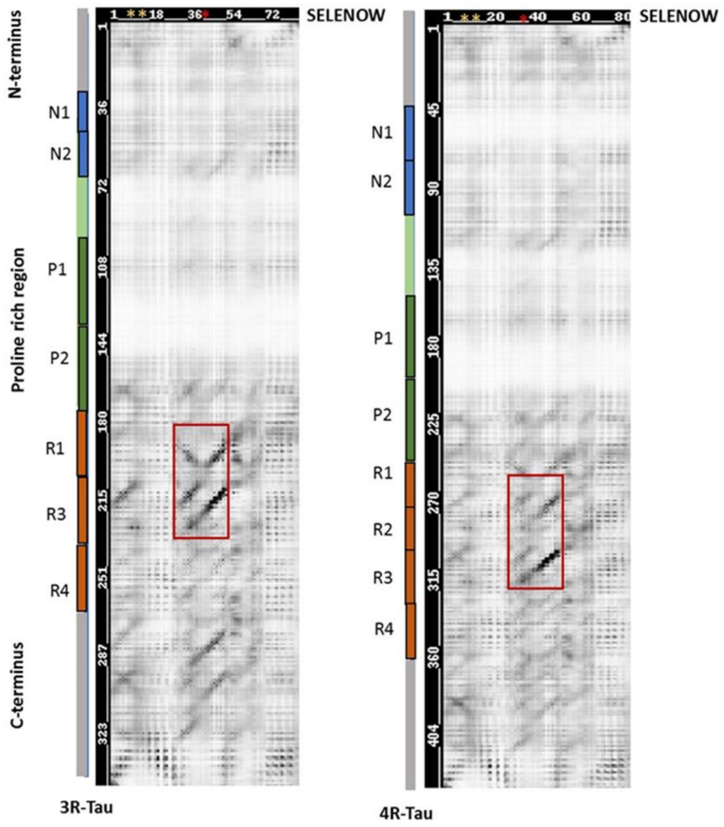

Supplementary Figure 6. The ComplexContact predicted interfacial contacts between SELENOW and 2N3R-Tau (410aa, left panel), and between SELENOW and full length 2N4R-Tau (441aa, right panel).

Supplementary Figure 7. Uncropped blots

Anti-HT7	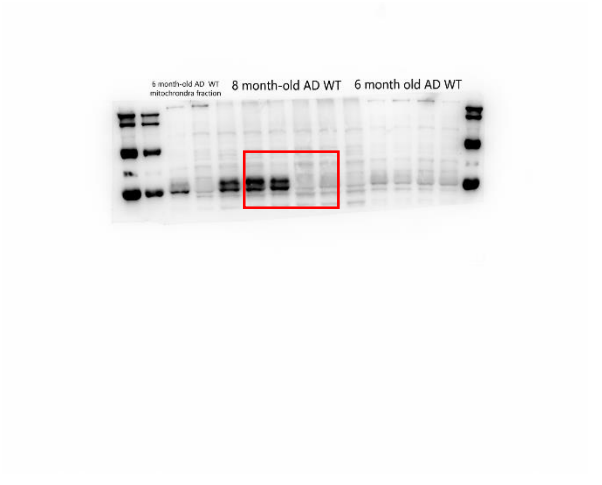
Anti-SELENOW	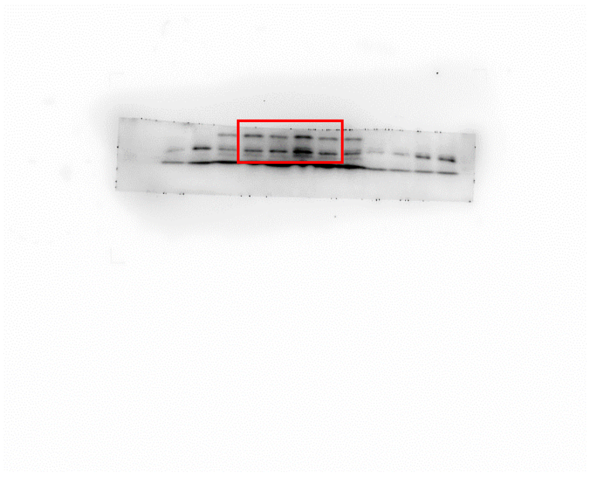
Anti-GAPDH	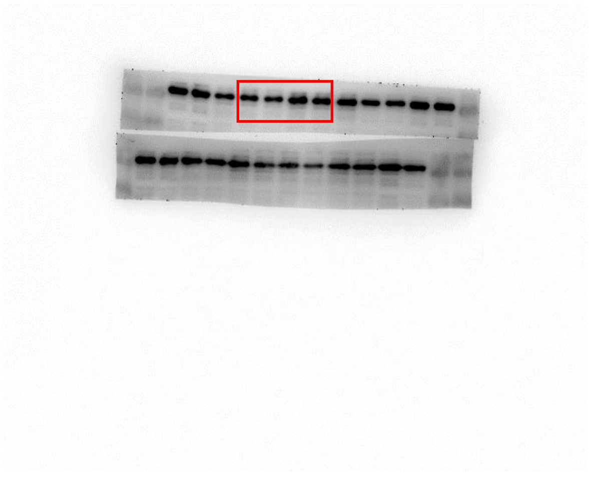

Anti-myc	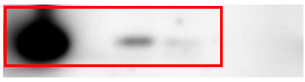

Anti-tau5	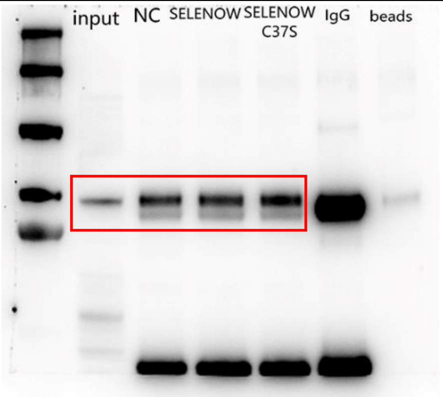

Anti-tau5	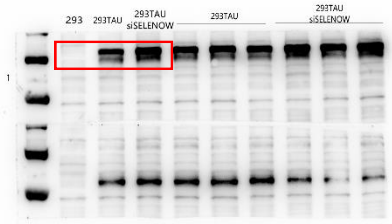
Anti-SELENOW	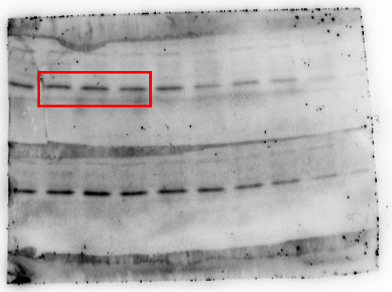

Anti-GAPDH	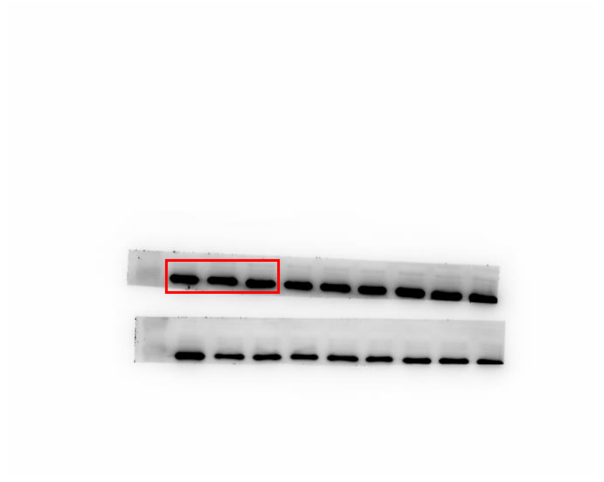

Anti-tau5	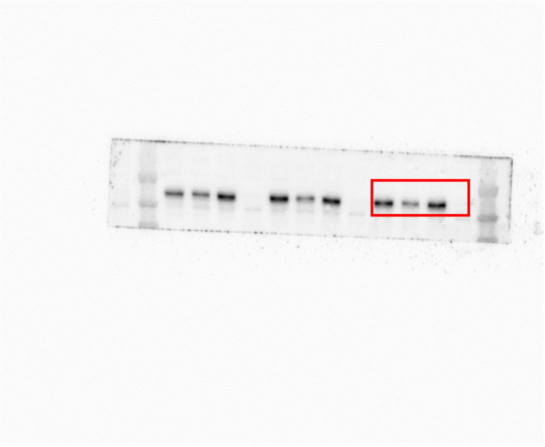
Anti-myc (SELENOW)	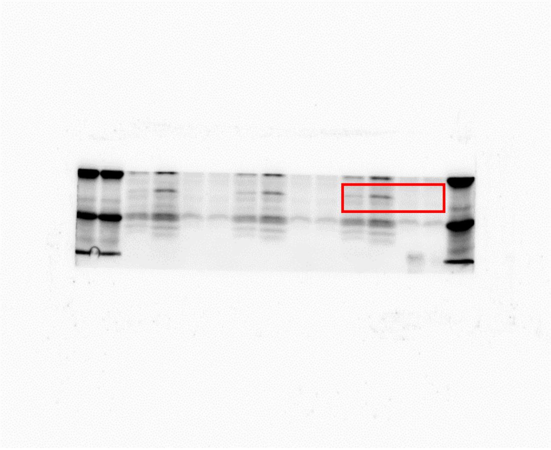

Anti-GAPDH	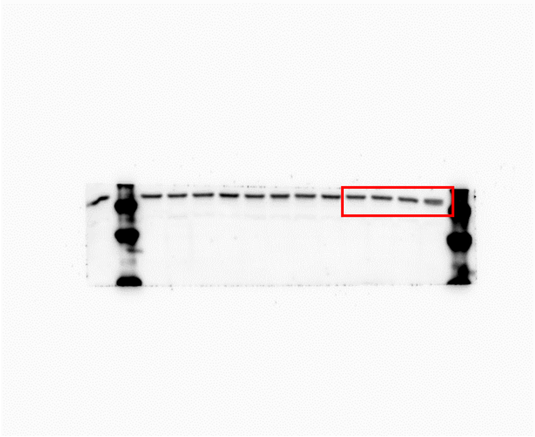
Anti-tau5	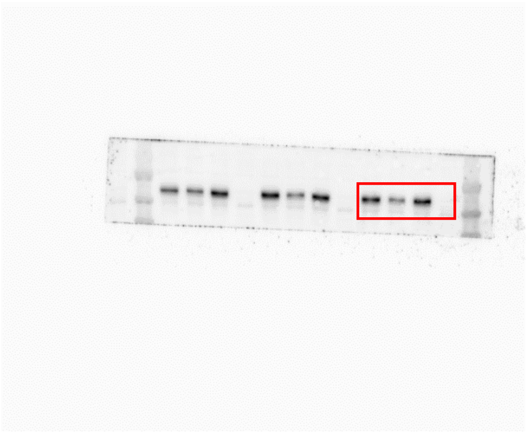
Anti-myc(SELENOV)	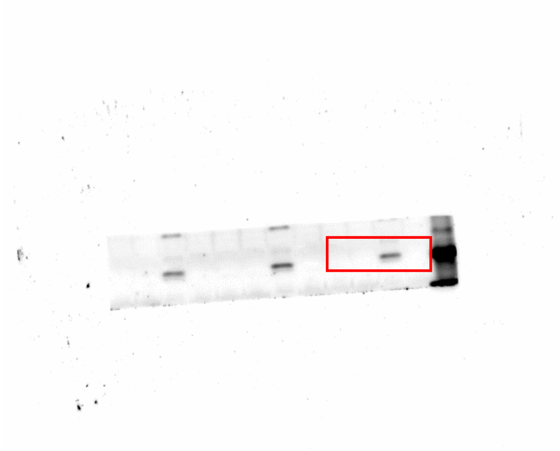

Anti-GAPDH	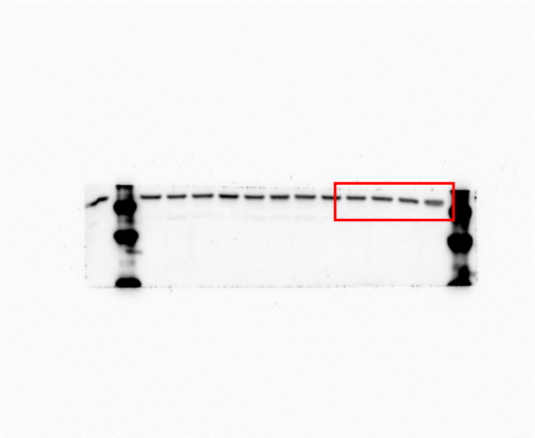
Anti-myc(SELENOW)	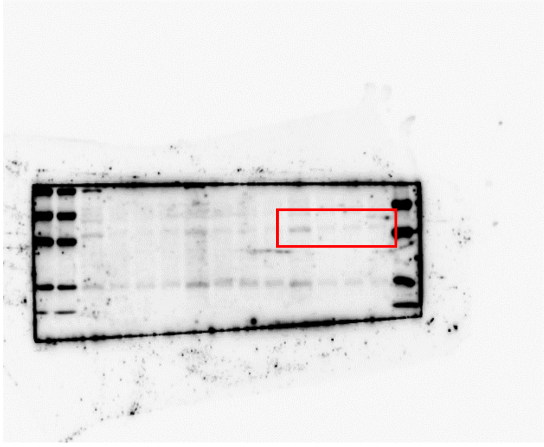

Anti-tau5	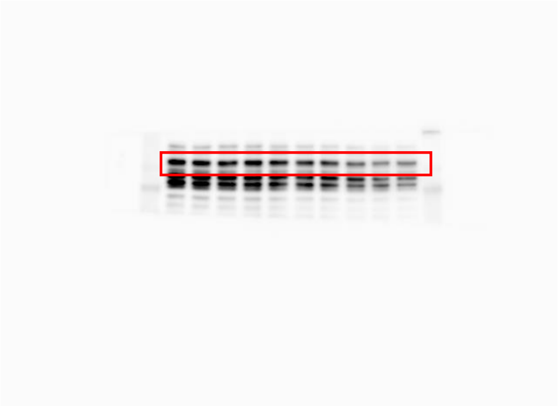
Anti-myc	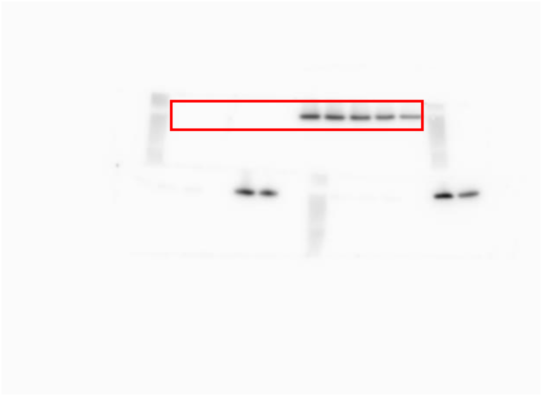

Anti-β-actin	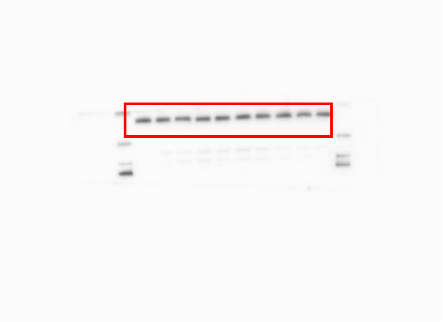

Anti-tau5<br>GAPDH-HRP	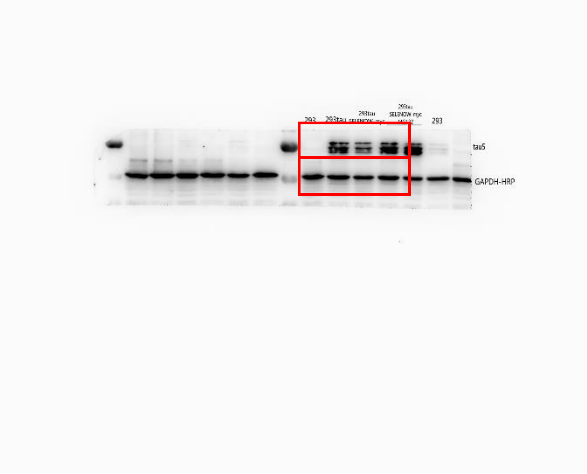
Anti-myc	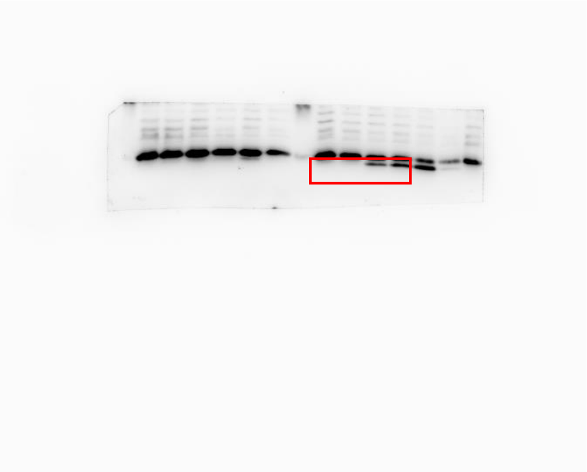

Anti-tau5	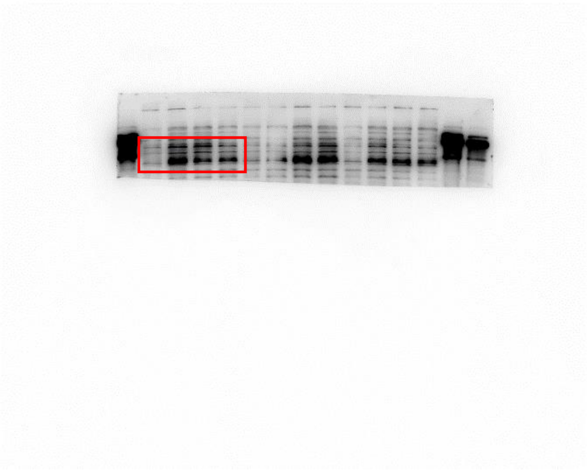
Anti-LC3A/B	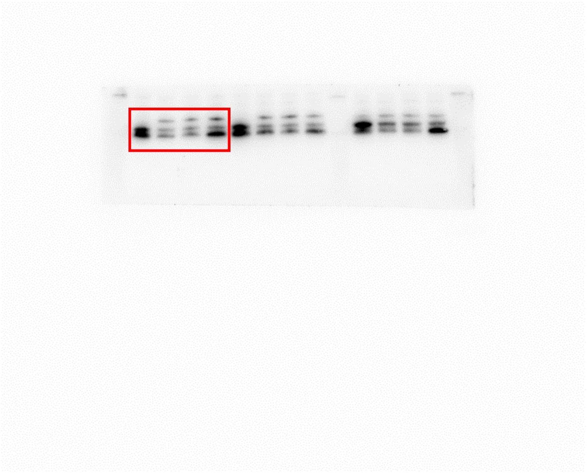
Anti-myc	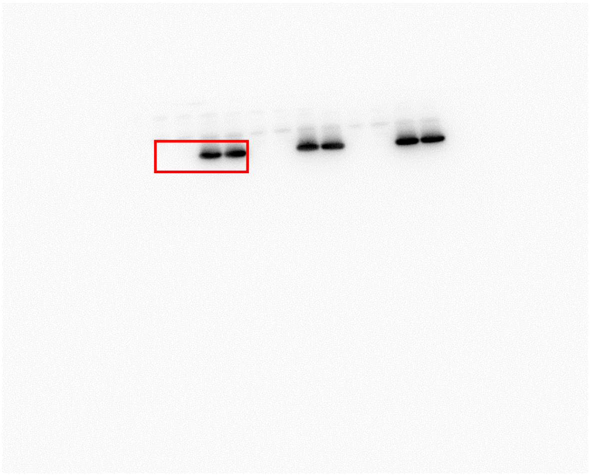

Anti-GAPDH	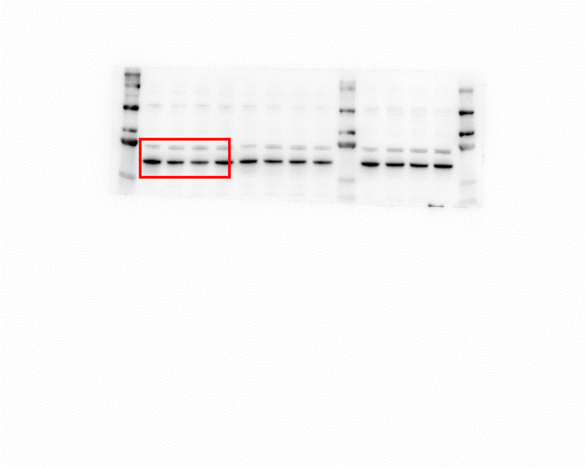

Anti-Hsp70	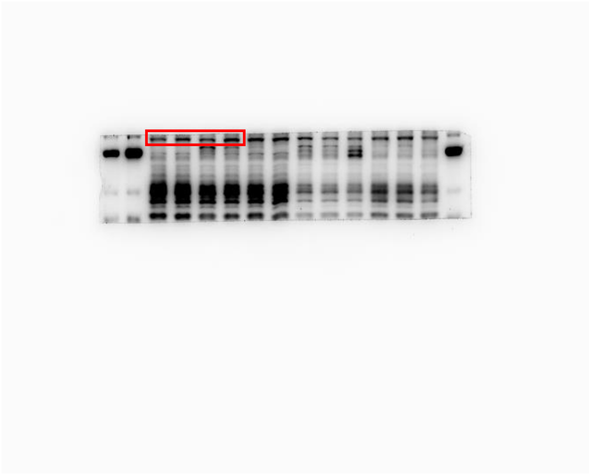
Anti-tau5	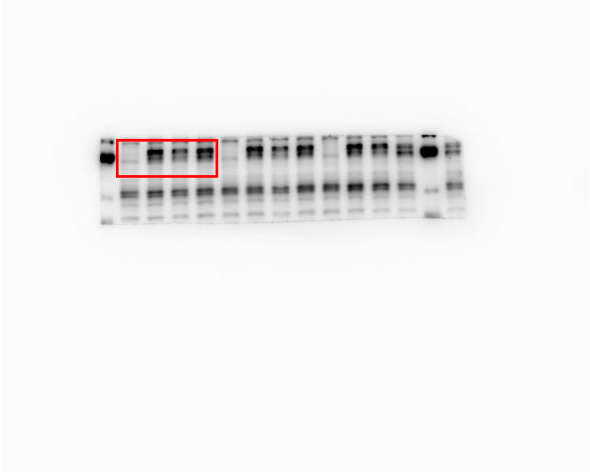

Anti-myc	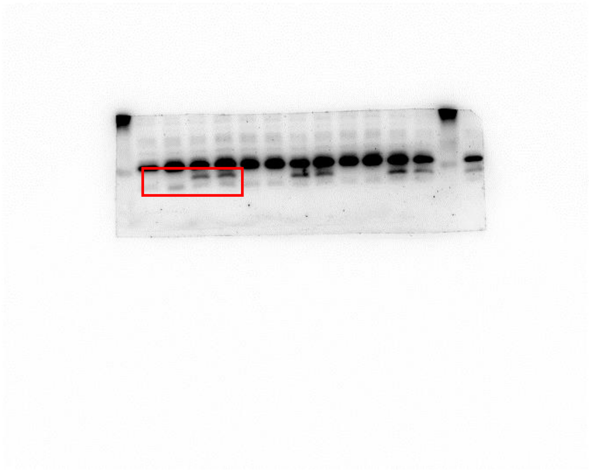
Anti-GAPDH	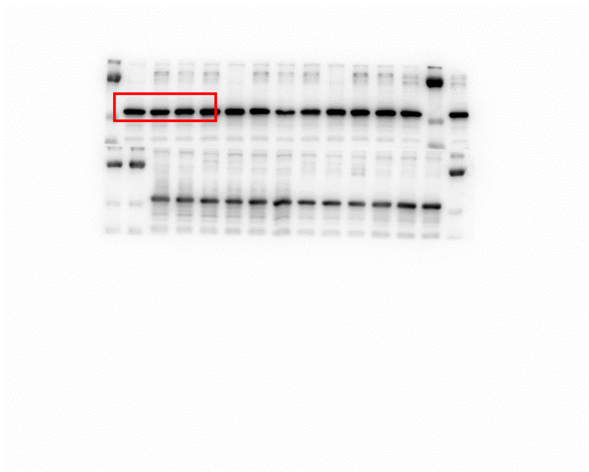

Anti-GFP	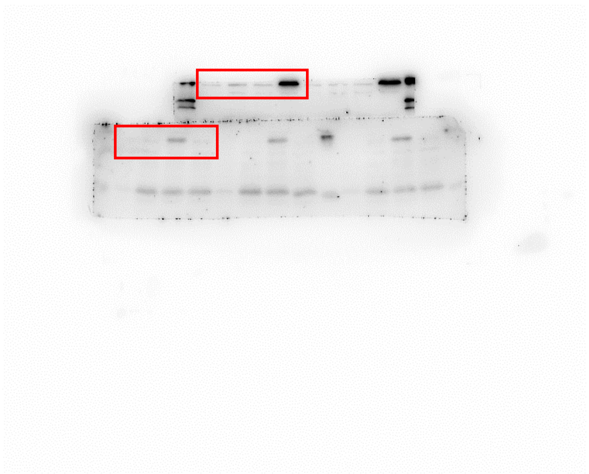

Anti-tau5	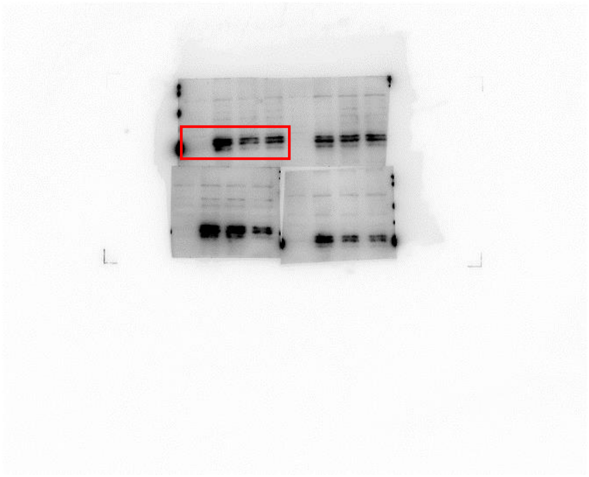
Anti-GAPDH	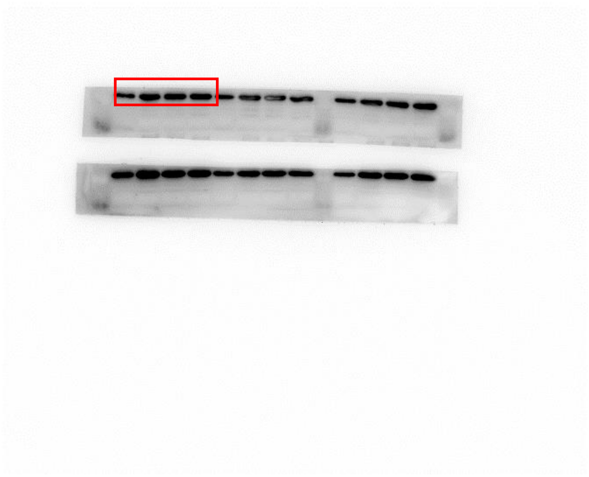
Anti-myc	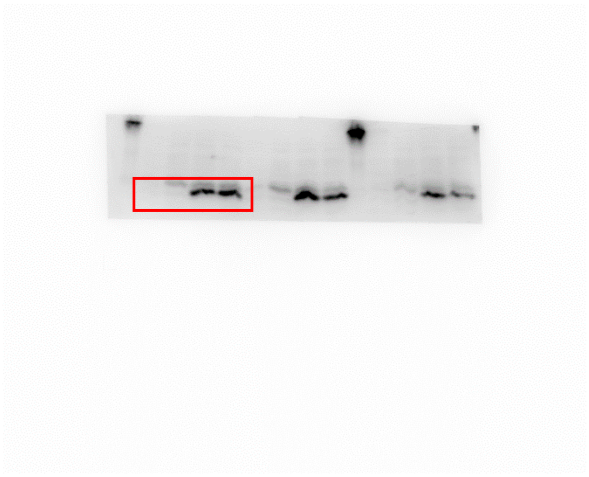

Anti-myc	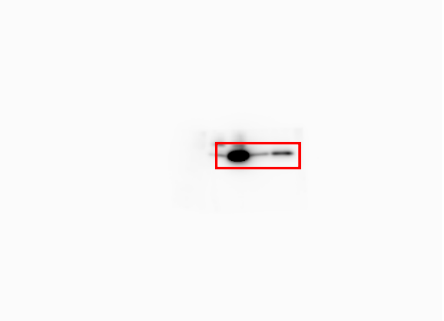
Anti-tau5	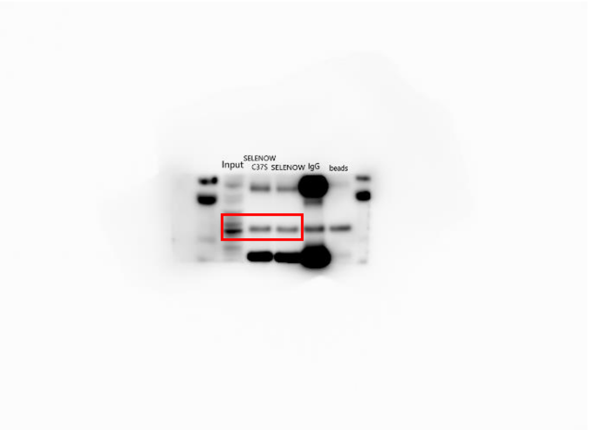
Anti-HSP70	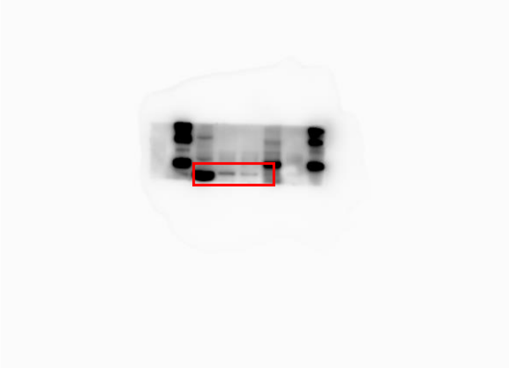
Anti-ubiquitin	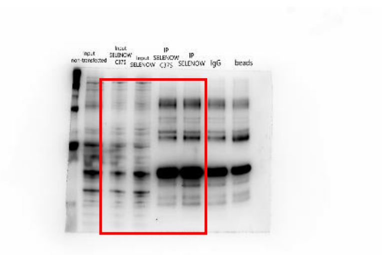

Anti-PSD95	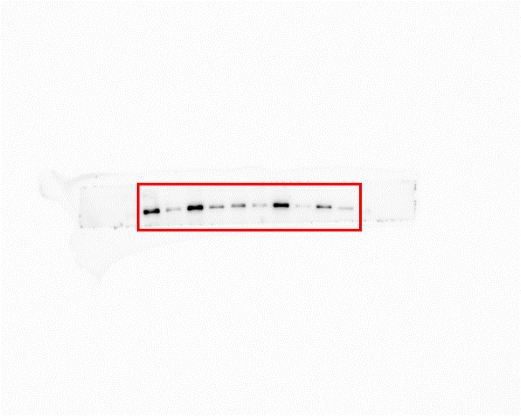
Anti-Syn	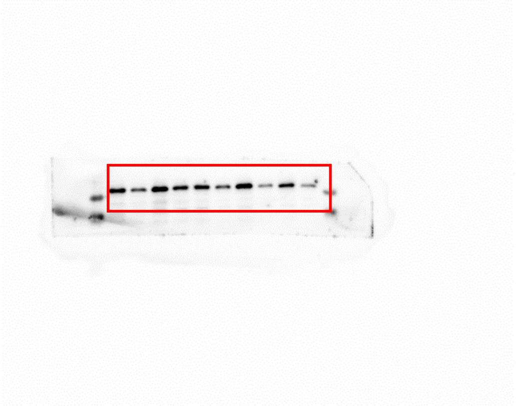
Anti-α-tubulin	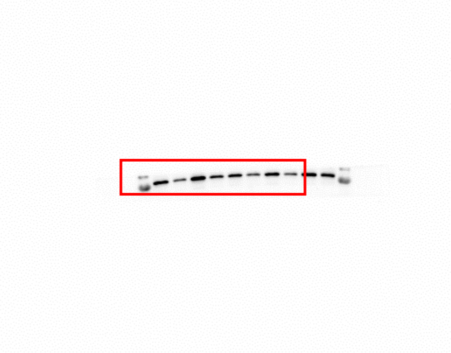
Anti-SELENOW	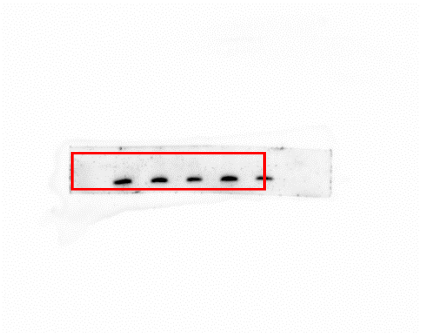

Anti-tau5	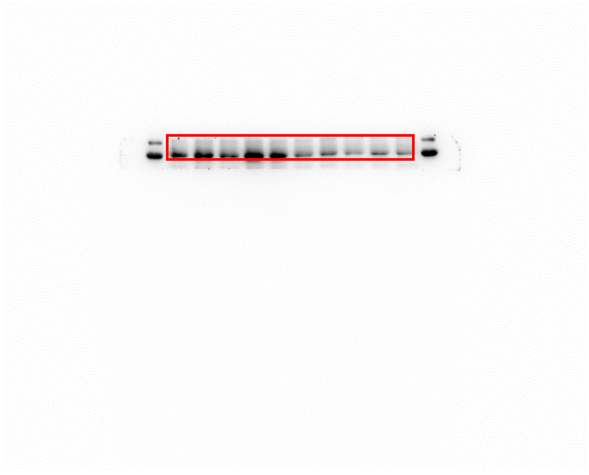
Anti-GAPDH	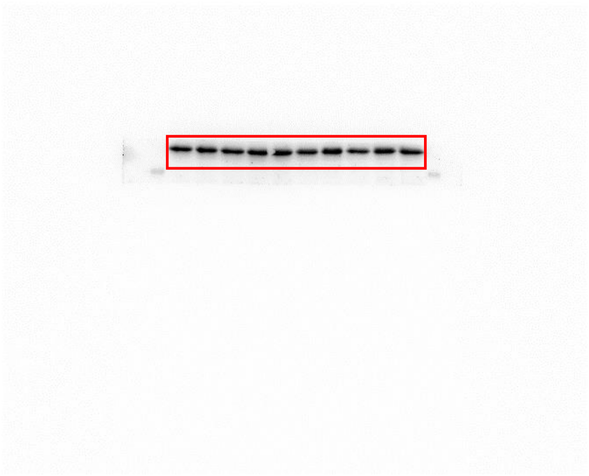

Anti-HT7	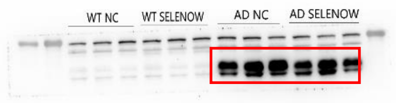

Anti-RD3	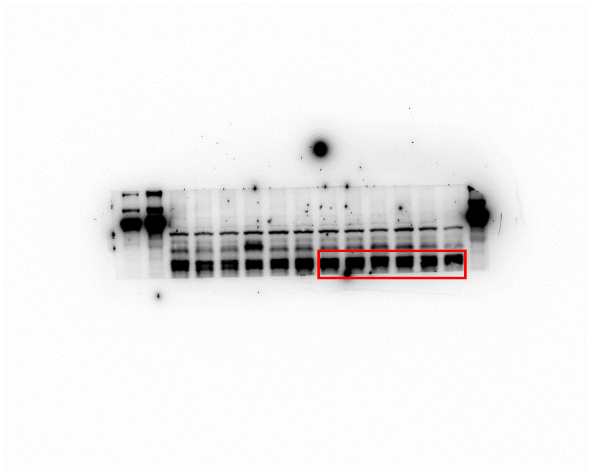
Anti-RD4	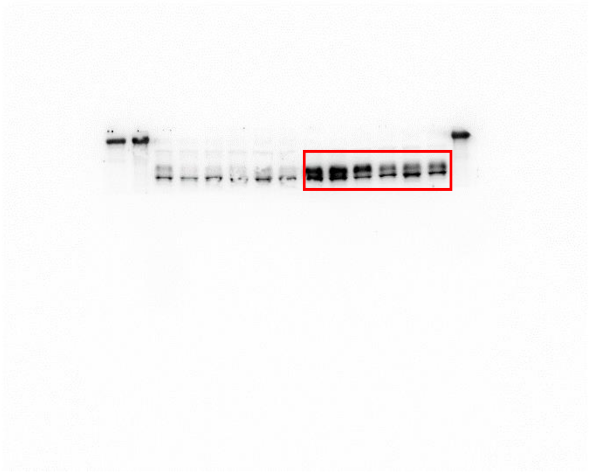
Anti-GAPDH	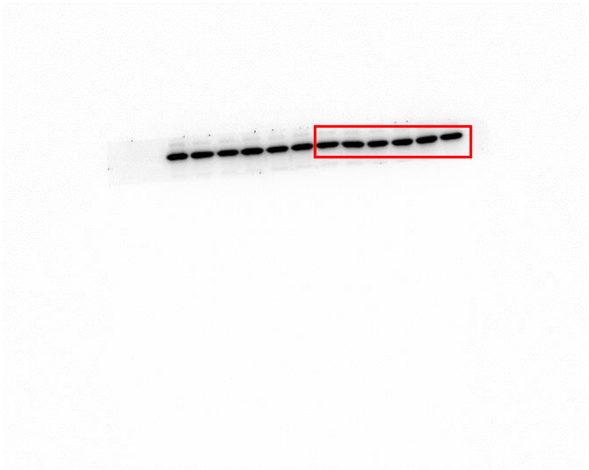

Anti-SELENOW	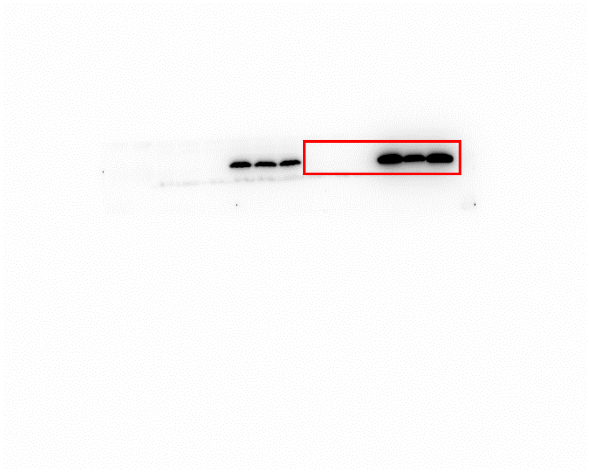
Anti-pTau181	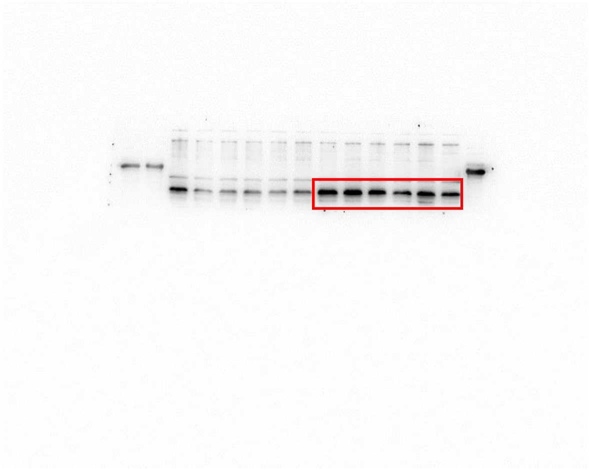
Anti-pTau231	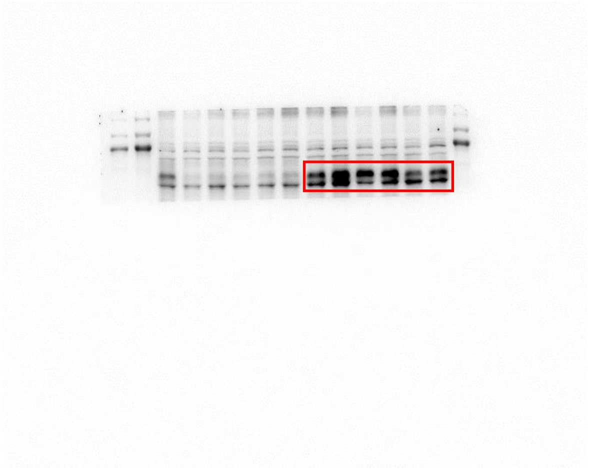

Anti-pTau396	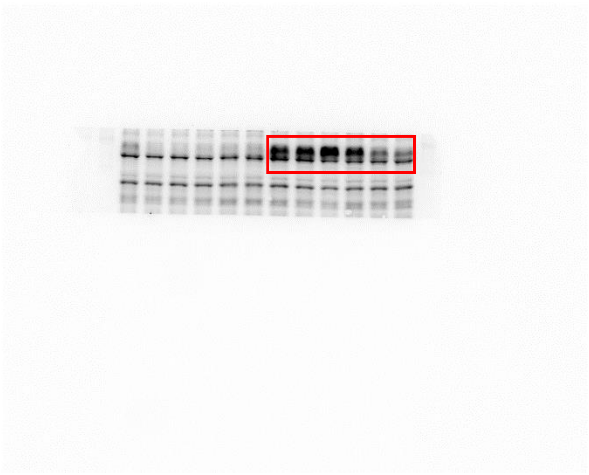
Anti-pTau422	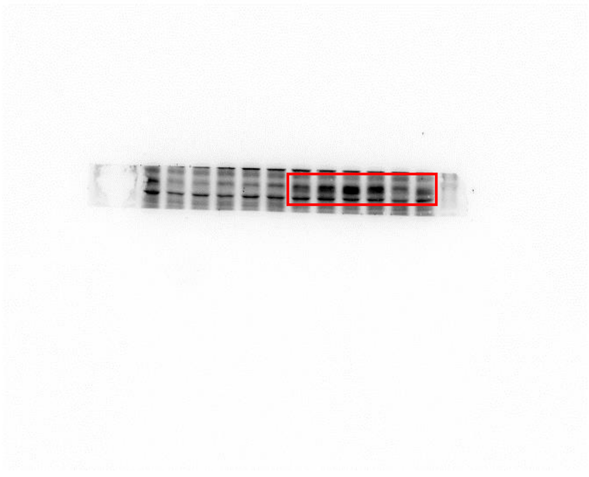
Anti-Tau5	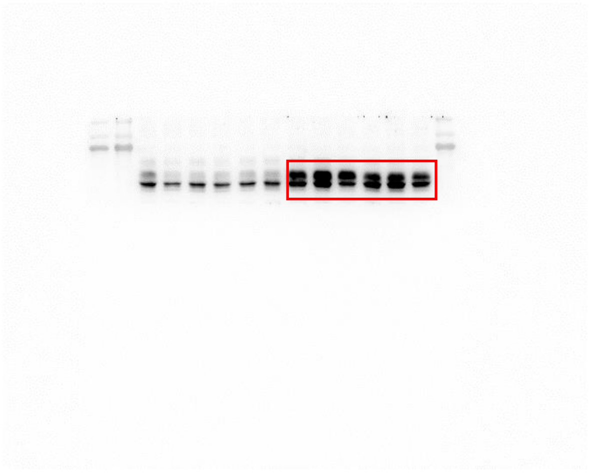

Anti-pTau202/205	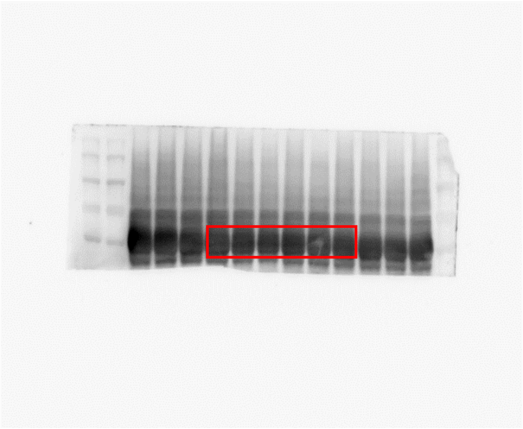
Anti-pTau262	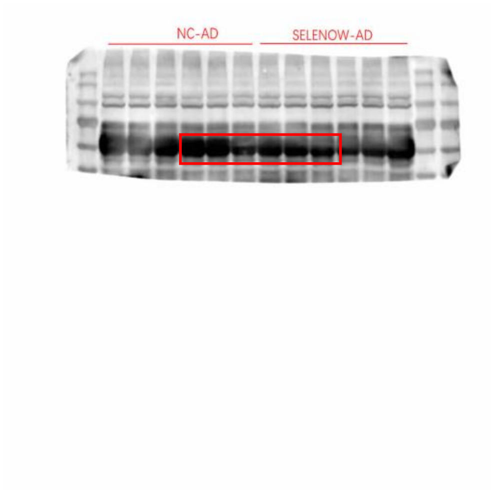
Anti-pTau404	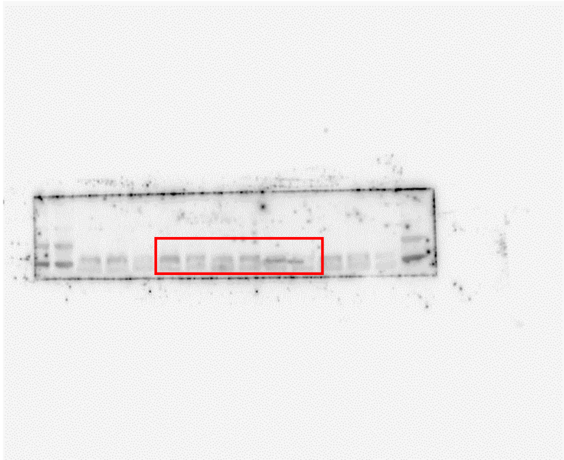

Anti-pTau416	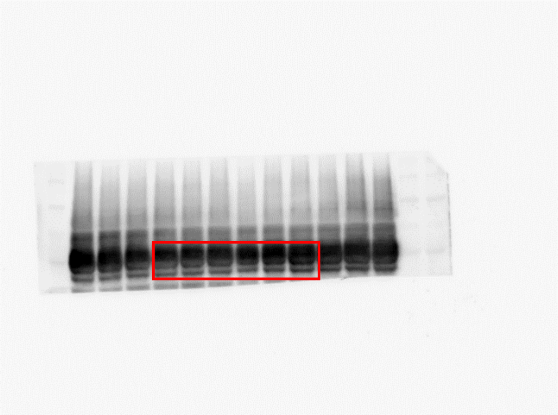
Anti-Tau5	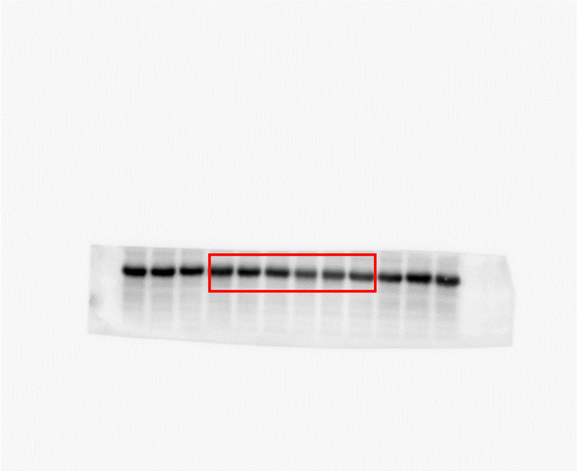

Anti-6E10	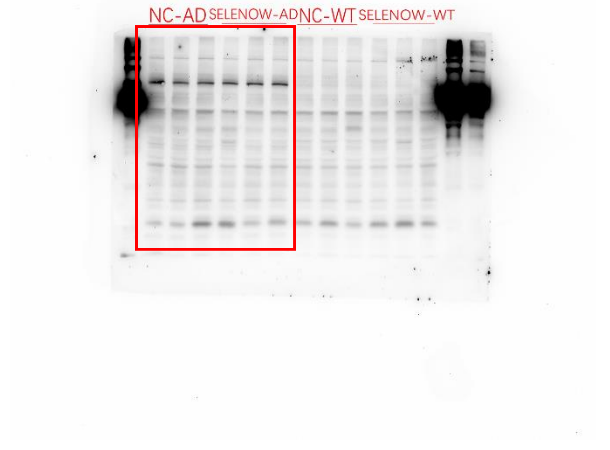

Anti-GAPDH	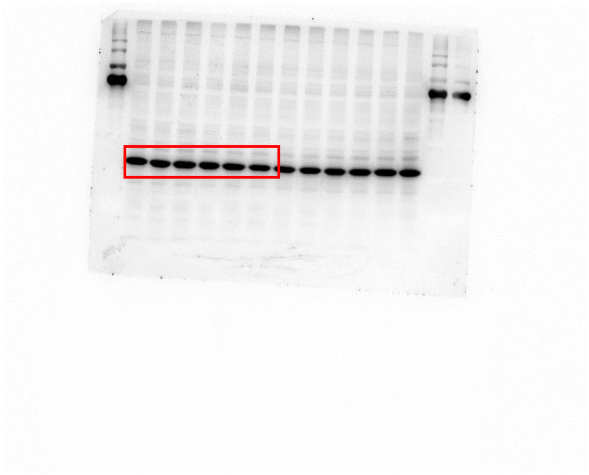
Anti-Iba1	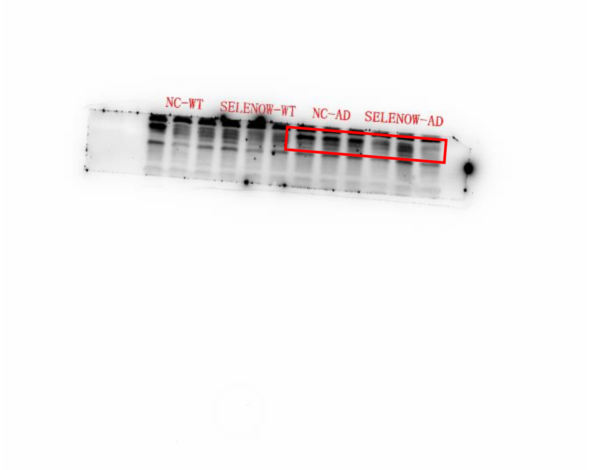
Anti-GFAP	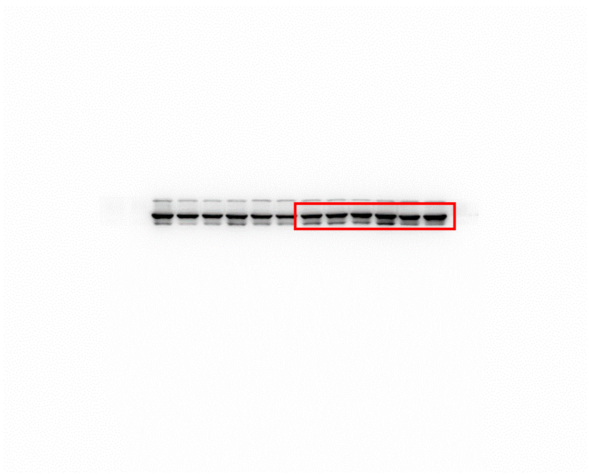

Anti-Oligo2	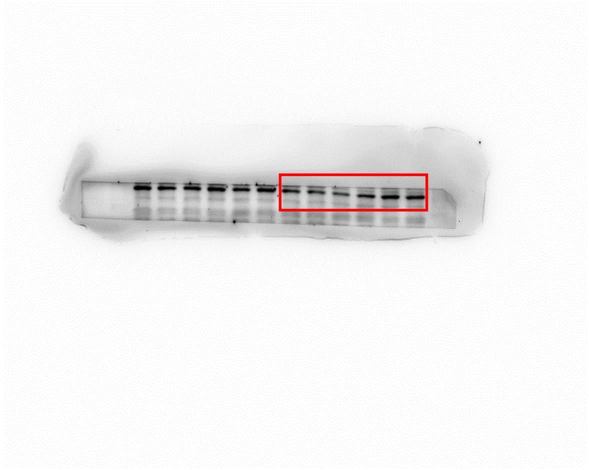
Anti-GAPDH	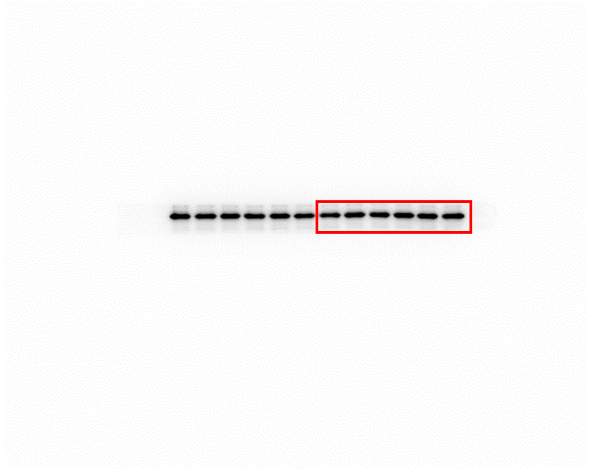

Supplementary Table 1

Q-PCR primers	
HSP90AB1	GTCTGGGTATCGGAAAGCAAG<br>CTGAGGGTTGGGGATGATGTC
HSPD1(HSP60)	TGCTGTTGAAGAAGGCATTG<br>CAGCAGCATCCAATA AAGCA
Hsc70(HSPA8)	CAGGTTTATGAAGGCGAGCGTGCC<br>GGGTGCAGGAGGTATGCCTGTGA
HSPA1A	AGAGCGGAGCCGACAGAG<br>CACCTTGCCGTGTTGGAA
Beta-actin	CCTGGCACCCAGCACAAT<br>GCTGATCCACATCTGCTGGAA
SELENOW (HUMAN)	ATGCCTGGACATTTGTGGCGA<br>GCAGCTTTGATGGCGGTCAC
SELENOW (MOUSE)	CTATAAGCCCAAGTACCTCCA<br>CTGTATCCACATAGCCATCAC
