# Supplementary material for: Selenoprotein W modulates tau homeostasis in an Alzheimer’s disease mouse model
Source: Commun Biol. 2024 Jul 17;7:872. doi: 10.1038/s42003-024-06572-0 (PMC11255228; doi:10.1038/s42003-024-06572-0)
Supplement: Supplementary file 4 — reporting summary [file 42003_2024_6572_MOESM4_ESM.pdf]

nature portfolio

Corresponding author(s): Qiong Liu

Last updated by author(s): Jun 21, 2024

Reporting Summary

Nature Portfolio wishes to improve the reproducibility of the work that we publish. This form provides structure for consistency and transparency in reporting. For further information on Nature Portfolio policies, see our Editorial Policies and the Editorial Policy Checklist.

Statistics

For all statistical analyses, confirm that the following items are present in the figure legend, table legend, main text, or Methods section.

n/a	Confirmed	
☐	☒	The exact sample size (*n*) for each experimental group/condition, given as a discrete number and unit of measurement
☐	☒	A statement on whether measurements were taken from distinct samples or whether the same sample was measured repeatedly
☐	☒	The statistical test(s) used AND whether they are one- or two-sided<br>*Only common tests should be described solely by name; describe more complex techniques in the Methods section.*
☒	☐	A description of all covariates tested
☒	☐	A description of any assumptions or corrections, such as tests of normality and adjustment for multiple comparisons
☒	☐	A full description of the statistical parameters including central tendency (e.g. means) or other basic estimates (e.g. regression coefficient) AND variation (e.g. standard deviation) or associated estimates of uncertainty (e.g. confidence intervals)
☒	☐	For null hypothesis testing, the test statistic (e.g. *F*, *t*, *r*) with confidence intervals, effect sizes, degrees of freedom and *P* value noted<br>*Give P values as exact values whenever suitable.*
☒	☐	For Bayesian analysis, information on the choice of priors and Markov chain Monte Carlo settings
☒	☐	For hierarchical and complex designs, identification of the appropriate level for tests and full reporting of outcomes
☒	☐	Estimates of effect sizes (e.g. Cohen's *d*, Pearson's *r*), indicating how they were calculated

Our web collection on statistics for biologists contains articles on many of the points above.

Software and code

Policy information about availability of computer code

Data collection	No software was used.
Data analysis	GraphPad Prism 8.0.2 (Statistical analysis)

For manuscripts utilizing custom algorithms or software that are central to the research but not yet described in published literature, software must be made available to editors and reviewers. We strongly encourage code deposition in a community repository (e.g. GitHub). See the Nature Portfolio guidelines for submitting code & software for further information.

Data

Policy information about availability of data

All manuscripts must include a data availability statement. This statement should provide the following information, where applicable:

- Accession codes, unique identifiers, or web links for publicly available datasets
- A description of any restrictions on data availability
- For clinical datasets or third party data, please ensure that the statement adheres to our policy

The data generated in the experiments is available from the authors upon request.

Research involving human participants, their data, or biological material

Policy information about studies with human participants or human data. See also policy information about sex, gender (identity/presentation), and sexual orientation and race, ethnicity and racism.

Reporting on sex and gender	We don't have related issues.
Reporting on race, ethnicity, or other socially relevant groupings	We don't have related issues.
Population characteristics	We don't have related issues.
Recruitment	We don't have related issues.
Ethics oversight	We don't have related issues.

Note that full information on the approval of the study protocol must also be provided in the manuscript.

Field-specific reporting

Please select the one below that is the best fit for your research. If you are not sure, read the appropriate sections before making your selection.

☒ Life sciences ☐ Behavioural & social sciences ☐ Ecological, evolutionary & environmental sciences

For a reference copy of the document with all sections, see nature.com/documents/nr-reporting-summary-flat.pdf

Life sciences study design

All studies must disclose on these points even when the disclosure is negative.

Sample size	We determined the number of experimental animals accordance to the references DOI: 10.7150/thno.17450: D0l: 10.7150/ibs.16150. Animal and cell n number were indicated in each bar graph overlaid with a dot plot .
Data exclusions	Outliers were detected using the Grubbs' test in PRISM and were excluded from the analysis of behavioral tests.
Replication	All attempts at replication were successful.
Randomization	The wildtype and 3×Tg?AD mice for brain SELENOW overexpression were randomly divided into two groups. The mice injected with AAV packed empty vector were used as the negative control group (NC-AD, NC-WT), and the mice injected with SELENOW(U13C) overexpression vector were defined as the experimental group (SELENOW-AD,SELENOW-WT) .
Blinding	The investigators were blinded to group allocation during data collection and analysis.

Reporting for specific materials, systems and methods

We require information from authors about some types of materials, experimental systems and methods used in many studies. Here, indicate whether each material, system or method listed is relevant to your study. If you are not sure if a list item applies to your research, read the appropriate section before selecting a response.

Materials & experimental systems

n/a	Involved in the study	
☐	☒	Antibodies
☐	☒	Eukaryotic cell lines
☒	☐	Palaeontology and archaeology
☐	☒	Animals and other organisms
☒	☐	Clinical data
☒	☐	Dual use research of concern
☒	☐	Plants

Methods

n/a	Involved in the study	
☒	☐	ChIP-seq
☒	☐	Flow cytometry
☒	☐	MRI-based neuroimaging

Antibodies

Antibodies used	Two different anti-tau protein antibodies, tau5 from Abcam (ab80579, monoclonal) and HT7 from Invitrogen (MN1000, monoclonal), were used in our experiments. For detection of tau phosphorylation at different epitopes, the anti-pTau181 from Abcam (ab75679,

polyclonal), anti-pTau 202/205 from Abclonal (AP1378, polyclonal), anti-pTau231 from Abcam (ab151559, monoclonal), anti-pTau262 from Abclonal (AP0397, polyclonal), anti-pTau396 from Abcam (ab109390, monoclonal), anti-pTau404 from Abclonal (AP1378, monoclonal), anti-pTau416 from Abclonal (AP1101, polyclonal) and anti-pTau 422 from Abcam (ab79415, monoclonal) were used in our experiments. For detection of different tau isoforms, the anti-Tau 4-repeat isoform RD4(# 05-804, monoclonal) and anti-Tau 3-repeat isoform RD3(#05-803, monoclonal) were purchased from Merck Millipore. The anti-SELENOW antibody was purchased from Rockland Immunochemical (600-401-A29, polyclonal). Anti-myc (2276, monoclonal), anti-GFP (2956, monoclonal), anti-Hsp70 (4872, polyclonal), anti-LC3A/B (12741, monoclonal), anti-ubiquitin (3936, monoclonal), and anti-acetylated lysine (9441, polyclonal) antibodies were purchased from Cell Signaling Technology (CST). For detection of oxidative stress, synaptic and glia protein markers, the anti- malondialdehyde (MDA, ab27642, polyclonal), anti-synaptophysin (ab32127, monoclonal) and anti-PSD95 (ab18258, polyclonal) were purchased from Abcam, anti-Iba1(17198, monoclonal) was purchased from CST, anti-Oligo2 (66513-1-lg, monoclonal) and anti-GFAP (16528-1-AP, polyclonal) were from Proteintech. Anti-GAPDH antibody was from ABclonal (A19056, monoclonal). Alexa Fluor 555-conjugated anti-mouse secondary antibodies and DAPI staining solution were obtained from CST. Peroxidase-conjugated anti-mouse/rabbit antibodies were from Abmart (m21001, m21002).

Validation

The validation of commercial antibodies used in this study were posted in manufacturer's website. The specificity of anti-SELENOW antibody was further tested with samples from our SELENOW knock-out mice (see https://doi.org/10.3390/antiox11050999).

Eukaryotic cell lines

Policy information about cell lines and Sex and Gender in Research

Cell line source(s)	HEK293 cell lines were purchased from National Collection of Authenticated Cell Cultures.
Authentication	None of the cell lines used were authenticated.
Mycoplasma contamination	All cell lines were tested negative for mycoplasma contamination.
Commonly misidentified lines (See ICLAC register)	No commonly misidentified lines was used.

Animals and other research organisms

Policy information about studies involving animals; ARRIVE guidelines recommended for reporting animal research, and Sex and Gender in Research

Laboratory animals	Triple-transgenic AD (Stock No.004807, 3×Tg�AD) mice homozygous for three AD-related mutant alleles (Psen1 M146V mutation, APPSwe and tauP301L) and background control mice (B6129SF2/J) were purchased from Jackson Laboratory. Homozygous SELENOW (Gene ID: 20364) knockout (referred to as SELENOW KO below) mice and wild-type (WT) mice (C57BL/6N) used as background controls were generated and bred by BIOCYTOGEN (Beijing, China).
Wild animals	The study did not involve wild animals.
Reporting on sex	The findings apply to both sexes.
Field-collected samples	The study did not involve samples collected from the field.
Ethics oversight	The use of animals was approved by the Ethics Committee of Shenzhen University.

Note that full information on the approval of the study protocol must also be provided in the manuscript.

Plants

Seed stocks	N/A
Novel plant genotypes	N/A
Authentication	N/A
